# Genome-wide association study of bronchopulmonary dysplasia: a potential role for variants near the *CRP* gene

**DOI:** 10.1038/s41598-017-08977-w

**Published:** 2017-08-24

**Authors:** Mari Mahlman, Minna K. Karjalainen, Johanna M. Huusko, Sture Andersson, M. Anneli Kari, Outi K. T. Tammela, Ulla Sankilampi, Liisa Lehtonen, Riitta H. Marttila, Dirk Bassler, Christian F. Poets, Thierry Lacaze-Masmonteil, Claude Danan, Christophe Delacourt, Aarno Palotie, Louis J. Muglia, Pascal M. Lavoie, Alice Hadchouel, Mika Rämet, Mikko Hallman

**Affiliations:** 1 PEDEGO Research Unit, Medical Research Center Oulu, University of Oulu, Oulu, Finland; 2 Department of Children and Adolescents, Oulu University Hospital, Oulu, Finland; 3 Children’s Hospital, University of Helsinki, and Helsinki University Hospital, Helsinki, Finland; 4 Tampere University Hospital, Tampere University, and Center of Pediatric Child Health, Tampere, Finland; 5 Department of Pediatrics, Kuopio University Hospital, Kuopio, Finland; 6 Turku University Hospital, and the University of Turku, Turku, Finland; 7 Department of Neonatology, University Hospital Zurich, and University of Zurich, Zurich, Switzerland; 8 Department of Neonatology, Tuebingen University Hospital, Tuebingen, Germany; 9 Department of Paediatrics, Cumming School of Medicine, University of Calgary, Alberta, Canada; 10 Inserm, U955, Créteil, France; 11 CRB, CHI-Creteil, France; 12 Department of neonatology, CHI-Creteil, Creteil, France; 13 AP-HP, Hôpital Necker-Enfants Malades, Service de Pneumologie Pédiatrique, Paris, France; 14 Université Paris-Descartes, Paris, France; 15 Analytic and Translational Genetics Unit, Department of Medicine, Massachusetts General Hospital, Boston, MA USA; 16 Program in Medical and Population Genetics, The Broad Institute of MIT and Harvard, Cambridge, MA USA; 17 The Stanley Center for Psychiatric Research, The Broad Institute of MIT and Harvard, Cambridge, MA USA; 18 Institute for Molecular Medicine Finland, University of Helsinki, Helsinki, Finland; 19 Psychiatric & Neurodevelopmental Genetics Unit, Department of Psychiatry, Massachusetts General Hospital, Boston, MA USA; 20 Department of Neurology, Massachusetts General Hospital, Boston, MA USA; 21 Perinatal Institute, Cincinnati Children’s Hospital Medical Center and Department of Pediatrics, University of Cincinnati College of Medicine, Cincinnati, OH USA; 22 BC Children’s Hospital Research Institute, Vancouver Canada, Vancouver, Canada; 23 BioMediTech Institute and Faculty of Medical and Life Sciences, University of Tampere, Tampere, Finland

## Abstract

Bronchopulmonary dysplasia (BPD), the main consequence of prematurity, has a significant heritability, but little is known about predisposing genes. The aim of this study was to identify gene loci predisposing infants to BPD. The initial genome-wide association study (GWAS) included 174 Finnish preterm infants of gestational age 24–30 weeks. Thereafter, the most promising single-nucleotide polymorphisms (SNPs) associated with BPD were genotyped in both Finnish (*n* = 555) and non-Finnish (*n = *388) replication cohorts. Finally, plasma CRP levels from the first week of life and the risk of BPD were assessed. SNP rs11265269, flanking the *CRP* gene, showed the strongest signal in GWAS (odds ratio [OR] 3.2, *p* = 3.4 × 10^−6^). This association was nominally replicated in Finnish and French African populations. A number of other SNPs in the *CRP* region, including rs3093059, had nominal associations with BPD. During the first week of life the elevated plasma levels of CRP predicted the risk of BPD (OR 3.4, *p* = 2.9 × 10^–4^) and the SNP rs3093059 associated nominally with plasma CRP levels. Finally, SNP rs11265269 was identified as a risk factor of BPD (OR 1.8, *p* = 5.3 × 10^−5^), independently of the robust antenatal risk factors. As such, in BPD, a potential role for variants near *CRP* gene is proposed.

## Introduction

Bronchopulmonary dysplasia (BPD) is a major complication of prematurity. In the USA alone, about 10,000–15,000 new cases are diagnosed each year^1^. The potential adverse consequences of BPD include asthma and chronic obstructive pulmonary disease (COPD)^2–4^. Effective prevention of BPD does not exist, and even with modern state-of-the-art therapies the incidence has not fallen^1^.

In BPD, poor alveolarization and disrupted pulmonary vascularization lead to impaired gas exchange, clinically seen as prolonged need for respiratory support^5^. Inflammation plays a key role in the pathogenesis of BPD, but the molecular mechanisms remain unknown^6, 7^. According to studies in twins, genetic factors account for 53–82% of the variance in susceptibility to BPD^8, 9^. However, identification of predisposing genes has been challenging. Although a suggestive association between *SPOCK2* gene and BPD was discovered in the first published genome-wide association study (GWAS) on BPD^10^, two subsequent GWASs did not reveal any significant associations at the genome-wide level^11, 12^. Moreover, candidate gene studies have been largely unsuccessful at producing replicable results^13, 14^. One probable reason for this “missing heritability” is the heterogeneity of study populations. In Hadchouel *et al*.’s study, the infants were of either Caucasian or African origin. Wang *et al*.’s population consisted of four identified ethnic groups, and Ambalavanan *et al*.’s study included both Caucasians and African-Americans. Different ethnic groups differ in their allele frequencies, thus complicating genetic analyses.

The population of Finland is genetically relatively homogeneous and has therefore been used extensively in genetic studies^15, 16^. Furthermore, neonatal practices vary little in Finland due to standardized treatment protocols. This is of importance when studying a trait such as BPD, the incidence of which is affected by neonatal treatment practices.

In the present investigation, we conducted a GWAS on BPD in a Finnish population. To make the results more generalizable, we studied two replication populations from Finland and two populations from Canada and France. We identified a single nucleotide polymorphism (SNP), rs11265269, as an independent risk factor of BPD.

## Methods

### Study design and study populations

**Figure 1:**
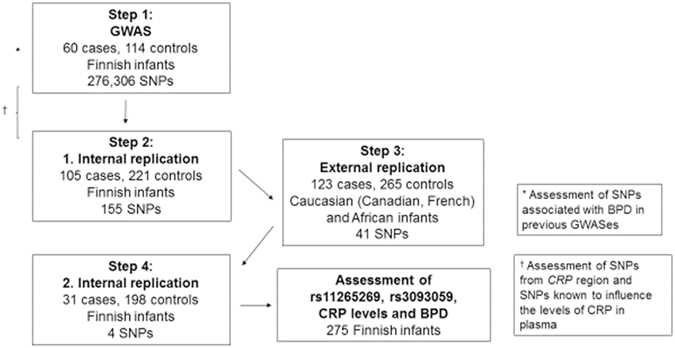
Flow chart of the study.

A flow chart of the study is shown in Fig. 1. The total numbers of BPD cases and controls in the entire study were 319 and 798, respectively.

Inclusion criteria for all infants included in the study were (1) gestational age (GA) <31 wk and (2) no major congenital malformations. The discovery population used for the GWAS (step 1 of the study, *n* = 174) and the two internal replication populations (first internal replication, step 2 of the study, *n* = 326 and second internal replication, step 4 of the study, *n* = 229) consisted of Finnish infants collected prospectively at the five university hospitals in Finland. The external replication populations (step 3 of the study, *n* = 388) comprised infants from Canada and France. To augment the power of the GWAS study, two control infants were chosen for each BPD case. Only one infant from each sibling pair was selected. For details of patient selection, see Supplementary Information. Clinical characteristics of the population in the GWAS are shown in Table 1, and those of the replication populations are shown in Supplementary Tables S1 and S2. The study was approved by the Ethics Committee of Oulu University Hospital, the University of British Columbia Clinical Research Ethics Board, the University of Alberta Ethics Board, and the Comités de Protection des Personnes (CPP) Ile de France VI and IX. Written informed consent was obtained from the infants’ parents. All experiments were performed in accordance with relevant guidelines and regulations.

**Table 1:** Clinical characteristics of the study population in the genome-wide association study of bronchopulmonary dysplasia.

Characteristics	BPD cases	Controls	*p*
Total *n*	60	114	
Moderate/severe BPD, *n* (%)	33/27 (55.0/45.0)		
No BPD/mild BPD, *n* (%)		64/50 (56.1/43.9)	
GA, weeks*^,†^	27.1 ± 1.8 (24.1–30.9)	27.5 ± 1.7 (23.3–30.9)	0.114
GA < 28 wk, *n* (%)	42 (70.0)	66 (57.9)	0.118
Birth weight, grams*	865 ± 244 (440–1470)	1017 ± 264 (520–1695)	2.85 × 10^–4^
Birth weight Z-score*^‡^	−1.5 ± 1.34 (−4.0–1.1)	−0.9 ± 1.20 (−4.1–1.6)	3.00 × 10^−3^
SGA, *n* (%)	23 (38.3)	20 (17.5)	3.00 × 10^−3^
Male gender, *n* (%)	32 (53.3)	57 (50.0)	0.676
Singletons, *n* (%)	57 (95.0)	105 (92.1)	0.474
Antenatal steroids, *n* (%)	57 (95.0)	108 (94.7)	0.941
Preeclampsia, *n* (%)	23 (38.3)	24 (21.1)	0.046
Chorioamnionitis^§^	15 (26.8)	38 (34.5)	0.311

*Definition of abbreviations*: BPD, bronchopulmonary dysplasia; GA, gestational age; SD, standard deviation; SGA, small for gestational age (Z-score ≤−2 SD).

*Mean ± standard deviation (range).

^†^GA defined on the basis of foetal ultrasound before 15 weeks of pregnancy.

^‡^Birthweight Z-score describes distribution of birthweight at given length of gestation in SD.

^§^Data not available for eight infants.

### Diagnostic criteria for BPD

BPD was defined as a continuing need for supplemental oxygen or positive pressure support (including high-flow 5–6 litre per minute via nasal probes) at 36 wks. postmenstrual age (PMA); i.e., moderate-to-severe BPD according to National Institute of Child Health and Human Development (NICHD) criteria^17^. This criterion was based on twin studies, in which moderate-to-severe BPD is significantly affected by genetic factors whereas mild BPD is not^9^. The controls were infants without BPD or with mild BPD. According to NICHD criteria, mild BPD is defined as a need for supplementary oxygen or positive pressure support at the age of 28 days but no longer at the age of 36 wks. PMA. No BPD is defined as no need for supplementary oxygen or positive pressure support at the age of 28 days.

### Statistical analyses and outline of other methods

Specific details of statistical analyses, genotyping, data processing, imputation and selection of SNPs for replication analyses, and analyses of CRP values, are shown in Supplementary Information. Information about DNA sample preparation is shown in Supplementary Table S3.

Genome-wide SNP genotyping was performed with the Infinium HumanCoreExome BeadChip (Illumina, San Diego, CA, USA). GWAS was used to identify SNPs associated with moderate-to-severe BPD; data processing and statistical analyses were performed with PLINK, v. 1.07 or 1.09^18^. After quality control, 276,306 SNPs with minor allele frequency >0.01 remained for GWAS. We used a filtering strategy to narrow down the SNPs analyzed for association with BPD in each step (Fig. 1): the most promising GWAS SNPs were analyzed in the internal Finnish replicate, and the most consistent SNPs were further analyzed in two external populations (Caucasian and French African). At the final step, four SNPs were analyzed in the second internal Finnish replicate. Logistic regression under the additive model with GA as a covariate was used to assess associations with BPD in these populations. The SNP selection criteria are listed in the Supplementary Information. At the final stage, we also included small-for-gestational age (SGA, Z-score ≤−2 SD) as a covariate in a logistic regression model to assess how the significant antenatal factors together (GA and SGA) with the SNP with most consistent signal affect the risk of BPD.

Because the SNP with most consistent signal was located near the *CRP* gene, we analyzed plasma CRP levels for association with BPD and SNP genotypes. Daily CRP levels were available because of the current practice in screening for infection during the first week after very premature birth. The infants included in the plasma CRP studies were a subset of the populations analyzed in the GWAS and the first internal replicate; infants for whom CRP levels could be recorded were included (*n* = 275; 112 infants from GWAS, 163 infants from first internal replicate). Logistic regression was used to evaluate whether SNP genotypes were associated with maximum and mean CRP level (CRP values above/below median) during the first week of life with PLINK, v. 1.09^18^. The number of surfactant doses was included as a covariate, because this variable associated with CRP levels (*p* < 0.001). Association of the BPD incidence or SNP genotypes with CRP levels was assessed by the *χ* ^2^ or Kruskal–Wallis test with SPSS Statistics 20.0, IBM Corporation. At the final phase, logistic regression with SPSS was used to evaluate whether CRP levels are significant predictors of BPD using GA and SGA as covariates.

## Results

### Associations in GWAS

The GWAS was performed in a discovery cohort of 60 cases with moderate-to-severe BPD and 114 controls. GA, gender, proportion of singletons, exposure to antenatal or postnatal steroids or to clinical chorioamnionitis did not differ between the cases and controls. As expected^19, 20^, both birth weight and birth-weight Z-score were lower in BPD cases compared with controls, suggesting a lower foetal growth rate among BPD infants. Pre-eclampsia was more common in the BPD group (Table 1).

In the GWAS, none of the SNPs reached our stringent genome-wide significance level (*p* < 1.8 × 10^−7^), but there were many suggestive associations (Supplementary Figure S1). Results for the SNPs with the strongest suggestive signals (*p* < 1 × 10^−4^) are shown in Table 2. The most promising SNP was rs11265269 (odds ratio [OR] 3.22, *p* = 3.43 × 10^−6^), which is located between the *CRP* and *DUSP23* genes (approximately 44 and 23 kb upstream of each gene, respectively). Population stratification was minimal (Supplementary Figure S2). The imputed SNPs did not show any association signals that were clearly more significant than those of the genotyped SNPs (Supplementary Figure S3). The SNPs with *p* < 5 × 10^−4^ in the GWAS were selected for further genotyping, and are listed in Supplementary Table S4.

**Table 2:** Single-nucleotide polymorphisms showing suggestive association signals in the genome-wide association study of bronchopulmonary dysplasia.

SNP information				GWAS		
SNP^*^	Chr	Position^†^	Gene^‡^	Case/Control minor allele frequency	OR	*p*
rs11265269	1	159,728,127	*CRP, DUSP23*	0.392/0.167	3.22	3.43 × 10^−6^
rs1481294	11	38,604,075	*LOC103312105, LOC105376635*	0.325/0.575	0.36	9.56 × 10^−6^
rs2351857	7	137,467,829	*DGKI*	0.617/0.373	2.71	1.42 × 10^−5^
rs11691168	2	74,999,114	*LOC102724482*	0.475/0.250	2.71	2.13 × 10^−5^
rs2149564	9	98,607,989	*LINC00476*	0.642/0.404	2.65	2.39 × 10^−5^
rs6562965	13	77,351,486	*LOC105370265*	0.442/0.224	2.75	2.42 × 10^−5^
rs11745686	5	74,198,611	*LOC105379039, FAM169A*	0.400/0.193	2.79	3.15 × 10^−5^
rs1403617	3	165,255,278	*LINC01322, BCHE*	0.475/0.254	2.65	3.20 × 10^−5^
rs12788032	11	38,632,770	*LOC103312105, LOC105376635*	0.275/0.504	0.37	3.89 × 10^−5^
rs1822471	15	79,327,227	*RASGRF1*	0.092/0.281	0.26	4.57 × 10^−5^
rs9552800	13	23,599,673	*LOC105370111, SGCG*	0.242/0.083	3.51	4.67 × 10^−5^
rs17537018	5	155,458,803	*SGCD, LOC105377674*	0.367/0.171	2.81	4.70 × 10^−5^
rs2527506	7	2,968,361	*CARD11*	0.325/0.140	2.95	4.85 × 10^−5^
rs4704970	5	155,500,992	*SGCD, LOC105377674*	0.358/0.167	2.79	5.78 × 10^−5^
rs1358603	7	52,757,480	*LOC105375280, LOC101928257*	0.617/0.390	2.51	5.78 × 10^−5^
rs4506388	23	130,210,648	*ARHGAP36*	0.534/0.281	2.94	6.13 × 10^−5^
rs9979500	21	48,029,698	*S100B, PRMT2*	0.450/0.241	2.57	6.57 × 10^−5^
rs4640066	13	77,354,747	*LOC105370265*	0.475/0.263	2.53	7.00 × 10^−5^
rs2352931	16	86,169,068	*LOC105376778, LOC101928582*	0.200/0.412	0.36	7.01 × 10^−5^
rs12603672	17	51,115,243	*LOC105371831, LOC101927337*	0.117/0.018	7.40	7.23 × 10^−5^
rs2279073	19	44,739,303	*ZNF227*	0.325/0.548	0.40	7.32 × 10^−5^
rs1044189	12	7,053,149	*RNU7-1*	0.383/0.189	2.67	7.57 × 10^−5^
rs7934284	11	38,577,786	*LOC103312105, LOC105376635*	0.608/0.386	2.47	7.67 × 10^−5^
rs200642524	8	10,470,709	*RP1L1*	0.067/0.000	—	8.00 × 10^−5^
rs11200206	10	123,635,883	*ATE1*	0.442/0.237	2.55	8.29 × 10^−5^
rs2543361	14	76,593,690	*GPATCHL2L, IFT43*	0.575/0.355	2.46	8.34 × 10^−5^
rs314277	6	105,407,662	*LIN28B*	0.275/0.110	3.08	8.35 × 10^−5^
rs4583363	18	65,162,006	*DSEL, LOC643542*	0.408/0.211	2.59	9.16 × 10^−5^
rs11178156	12	70,635,747	*LINC01481, CNOT2*	0.258/0.474	0.39	9.67 × 10^−5^

*Definition of abbreviations*: BPD, bronchopulmonary dysplasia; Chr, chromosome; GWAS, genome-wide association study; OR, odds ratio; SNP, single-nucleotide polymorphism.

*SNPs with *p* < 1 × 10^−4^ in GWAS are shown.

^†^Chromosomal positions refer to human genome build 37 (GRCh37/hg19).

^‡^Respective locus shown for SNPs within genes; two nearest loci shown for intergenic SNPs.

### Association of rs11265269 with BPD in replication populations

First, we chose 155 SNPs for analysis in the 1^st^ internal replication population (105 cases and 221 controls) (Supplementary Table S1). These SNPs were selected on the basis of the strongest signals in GWAS; in addition, previously BPD-associated SNPs with consistent signals were included. The selection criteria are given in detail in Supplementary Information. Thereafter, the top 41 SNPs, based on GWAS results and 1^st^ internal replication, were further analysed in the external replication populations (Caucasian, *n* = 312, and French African, *n* = 76) (Supplementary Table S2 and Supplementary Information). Finally, to increase power and to validate the most promising associations, SNPs rs11265269 (the top SNP identified by GWAS, near *CRP/DUSP23*), rs1889268 (*COL15A1*) and rs5999125 (*LARGE*) were genotyped in a second internal replication population (Finnish, *n* = 229). Of all the SNPs analysed in the external replication populations, these three SNPs showed the most consistent results and were thus selected for this final genotyping step. Because GA was significantly different between cases and controls in the replication populations, the analyses were performed using logistic regression (LR) analysis with GA as a covariate.

In the first internal replicate (Supplementary Table S4), only one SNP, rs6690148, showed nominal association with BPD (*p * < 0.05). The top SNP in GWAS, rs11265269 showed an effect similar to that detected in GWAS (OR 1.42, *p* = 0.097). Of the SNPs analysed further, two showed nominal association in the external replication populations: rs11265269 in the French African population and *COL15A1* SNP (rs1889268) in the Caucasian population (Supplementary Table S5). Overall, SNP rs11265269 (the SNP with the strongest signal in GWAS) was the most consistent SNP. Table 3 summarizes the results for this SNP in all the replication populations. In addition to the nominal replication in the French African population, this SNP associated with BPD when the internal replication populations were combined (*p* = 0.029). With all of the Finnish populations combined, the OR for the minor allele of SNP rs11265269 was 1.84 (*p* = 2.39 × 10^−5^) (Table 3); i.e., the effect was smaller than in the initial GWAS, which may be due to overestimated effect size in GWAS. The case–control allele frequency differences for the *COL15A1* SNP (rs1889268) were also consistent in all Caucasian populations, although the difference was not significant in the internal replication populations (Supplementary Tables S5 and S6). The results for the *LARGE* SNP (rs5999125) were more inconsistent (Supplementary Tables S5 and S6). The results for the other SNPs analysed in the replication populations are listed in Supplementary Tables S4 and S5.

**Table 3:** Association of single-nucleotide polymorphism rs11265269 with moderate-to-severe bronchopulmonary dysplasia.

Study population	*n*	Case/control minor allele frequency^*^	OR (95% CI)	*p*
Internal replication population 1 (Finnish)	326	0.278/0.230	1.42 (0.94–2.13)^†^	0.097
Internal replication population 2 (Finnish)	229	0.274/0.204	1.50 (0.75–2.86)^†^	0.216
Internal replication populations combined	555	0.277/0.218	1.47 (1.04–2.06)^†^	0.029
All Finnish populations combined	729	0.313/0.207	1.84 (1.39–2.45)^†^	2.4 × 10^–5^
External replication population 1 (Caucasian)	312	0.263/0.259	0.90 (0.59–1.37)^†^	0.629
External replication population 2 (French African)	76	0.440/0.235	2.48 (1.17–5.24)^†^	0.017

*Definition of abbreviations*: CI, confidence interval; OR, odds ratio.

^*^ Minor allele frequencies of the controls are similar to those of the populations of the 1000genomes project populations (0.239 and 0.223 for the European and African populations, respectively; http://www.1000genomes.org).

^†^Odds ratio for minor allele under additive model in logistic regression analysis with gestational age as a covariate.

We reanalysed the three SNPs with the strongest signals (rs11265269, rs1889268 and rs5999125) in the Finnish populations by excluding the mild BPD infants from the controls. This exclusion did not notably alter the results (Supplementary Table S7). For rs11265269 the OR was 3.28 in GWAS (*p* = 5.94 × 10^−5^) and 1.79 in the joint analysis of all Finnish populations (*p* = 1.41 × 10^−3^). Altered treatment practices or changes in the diagnostics of BPD (Supplementary Information) could also influence the results. Therefore, the genetic data was reanalysed separately for infants born during 1998–2010 and after 2010. During both time periods the association of rs11265269 with the risk of BPD was maintained in GWAS (OR 4.78 and 2.37, respectively) and in joint analysis of all Finnish populations (OR 1.87 and 1.71, respectively).

### SNPs previously associated with BPD: no clear association signals

Next, we analysed, first in our GWAS data, and thereafter the most promising SNPs in the internal replication population, the SNPs in proximity to genes that were previously found to be associated with BPD^10, 11^. Some of these SNPs showed a nominal association in the GWAS, but none were associated with BPD in the first internal replication population (Supplementary Tables S8 and S9). Consistently, none of the SNPs in previously-associated genes that were analysed further associated with BPD in the Caucasian and French African external replication populations (Supplementary Table S10). Overall, SNP rs2536512 in *SOD3* was the most promising. This SNP associated nominally with BPD in GWAS (OR = 0.53, *p* = 0.017), and showed a similar effect in the first internal replicate (OR = 0.75, *p* = 0.173) and external Caucasian population (OR = 0.70, *p* = 0.072). SNP rs2536512 reached *p* < 0.05 when the Caucasian population was combined with the internal replication population. However, there was no association in the French African replicate or in the second internal replicate (Supplementary Table S10). With all the Finnish populations combined, this SNP had an OR of 0.76 (*p* = 0.060).

### Evaluation of BPD risk and SNPs in the *CRP* region and in other regions previously associated with plasma CRP levels

**Figure 2:**
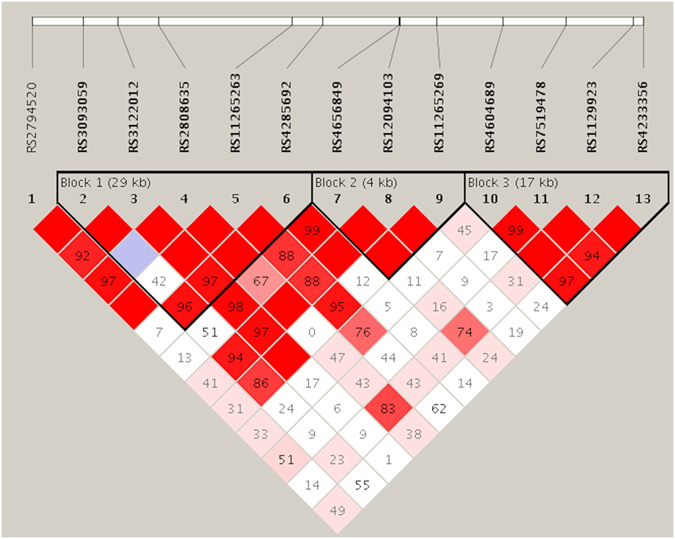
Linkage disequilibrium plot for single-nucleotide polymorphisms (SNPs) within the region flanking rs11265269, the top SNP in genome-wide association study (GWAS) of bronchopulmonary dysplasia. Pairwise *D*′ values for each SNP pair in the combined Finnish population (discovery GWAS and first internal replicate) are shown. Darker colors indicate stronger linkage disequilibrium. Of the SNPs analysed, rs2794520 and rs3093059 (*D*′ 0.86 with rs11265269) are located down- and upstream of the *CRP* gene, respectively; these SNPs are known to be associated with plasma levels of CRP. SNP rs1129923 is located within the *DUSP23* (dual specificity phosphatase 23) gene; rs7519478 and rs4233356 are up- and downstream of *DUSP23*.

Because SNP rs11265269 showed the most promising association with BPD and is located between *DUSP23* and *CRP* genes, we investigated this genomic region in more detail. We determined that rs11265269 displays linkage disequilibrium (LD) with some of the SNPs within or flanking the CRP gene rather than those flanking the DUSP23 gene (Supplementary Figure S4, Fig. 2). This is consistent with 1000 Genomes data (http://www.1000genomes.org). Furthermore, some of the other SNPs in the vicinity of only *CRP* gene also showed association signals in GWAS. Because many SNPs in this region are known to be associated with serum CRP levels, we further analysed 13 SNPs in the region extending from 69 kb 5′ to 3 kb 3′ of *CRP* to capture all common variations within this region in the first internal replication population (Supplementary Table S11). In addition to rs11265269, two SNPs (rs3093059 and rs12091403) in this region showed *p* < 0.05 for associations with BPD when the first internal replication population was combined with the population analysed in the GWAS. Haplotype analysis in this region revealed that three haplotypes were associated with BPD (*p* < 0.05); these could be discriminated by the three SNPs with *p* < 0.05 in the single SNP analyses (Table 4, Fig. 2). SNP rs11265269 was the only one of these three SNPs nominally associated with BPD in the Finnish and French African replication populations (Supplementary Table S12).

**Table 4:** Haplotype analysis within the region flanking rs11265269, the top single-nucleotide polymorphism (SNP) in genome-wide association study (GWAS) of bronchopulmonary dysplasia (BPD).

Haplotype block^*^	Haplotype	Case/control haplotype frequency^†^	*p*
1	AGACG	0.335/0.355	0.522
	AACCA	0.223/0.202	0.444
	AAACG	0.146/0.186	0.122
	AAACA	0.110/0.139	0.193
	AAAAG	0.091/0.064	0.129
	GAACA	0.092/0.049	8.2 × 10^–3^
2	GAA	0.385/0.473	8.8 × 10^−3^
	AGA	0.281/0.308	0.383
	GGG	0.324/0.210	9.1 × 10^−5^
3	AAGG	0.372/0.426	0.100
	GGGA	0.252/0.232	0.499
	GAAG	0.134/0.129	0.809
	GGGG	0.137/0.123	0.529
	GAGG	0.090/0.082	0.660

^*^Haplotype blocks are illustrated in Fig. 2. Block 1 consists of SNPs rs3093059, rs3122012, rs2808635, rs11265263, and rs4285692 adjacent to the *CRP* gene. Block 2 consists of SNPs rs4656849, rs12094103, and rs11265269. Block 3 consists of SNPs rs4604689, rs7519487, rs1129923, and rs4233356 encompassing the *DUSP23* gene. BPD-predisposing alleles of SNPs rs3093059 and rs11265269 and protective allele of rs12091403 are underlined; they discriminate the haplotypes showing association (*p* < 0.05).

^†^Frequency of haplotype in BPD cases and controls in the combined Finnish population (discovery GWAS and first internal replicate).

Using our GWAS data, we also analysed 108 SNPs (23 SNPs near *CRP* and 85 SNPs in other genes) that were previously reported to be associated with CRP levels^21–23^. Some of the SNPs had nominal associations with BPD (*p* < 0.05). However, in addition to those in the *CRP* gene region (Supplementary Table S11), none of them fulfilled our criteria to be included for genotyping in the replication sets.

### SNPs in the *CRP* gene region and plasma CRP levels

Next, we analysed whether SNPs near the *CRP* gene are associated with plasma CRP levels during the first week of life. Altogether, 275 Finnish infants were included. CRP values were obtained from clinical laboratory reports. Infants carrying the BPD-predisposing alleles of SNPs rs11265269 and rs3093059 tended to have higher CRP levels than non-carriers. In LR analysis with the number of surfactant doses as the established significant covariate, SNP rs3093059 was associated with mean plasma CRP concentrations (*p* = 0.046) but not with maximum plasma CRP concentrations (*p* = 0.074) (Supplementary Table S13) during the first week of life. SNP rs11265269 did not show association.

### Plasma CRP levels during the first week of life associated with BPD

Because the SNP with the strongest association signal in GWAS was located upstream of the *CRP* gene and other SNPs near *CRP* (including rs3093059) associated with plasma CRP levels in adult populations^22, 23^, and also showed nominal associations with BPD, we analysed whether maximum or mean CRP levels during the first week of life could predict development of BPD. Available laboratory data from 275 Finnish preterm infants were studied.

Plasma levels of CRP before 12 hours’ age were mostly low; only 8.4% of the infants had a CRP concentration of more than 1 mg/L; the maximum was 53.4 mg/L. These plasma levels were not associated with the risk of BPD (*p* = 0.66). We next studied whether maximum and mean plasma CRP concentrations during the 1^st^ week were associated with the risk of BPD. The median values of the maximum and mean CRP levels during the first week were 4.0 mg/L and 1.6 mg/L, respectively. The maximum plasma CRP concentration was above the median in 72.6% of the BPD cases and in 40.6% of the controls (*p* = 3.0 × 10^−6^). Mean CRP levels during the 1^st^ week of life exceeded the median in 74.0% of the BPD cases and 41.6% of the controls (*p* = 2.0 × 10^−6^) (Table 5). The number of surfactant doses given was associated with these CRP levels (*p* = 8.86 × 10^−8^ for the maximum and *p* = 3.25 × 10^−8^ for the mean level). In contrast, the length of gestation, prolonged rupture of membranes, chorioamnionitis, cord blood pH or blood culture-proven sepsis (either early- or late-onset during the first week) showed no detectable association.

**Table 5:** Association of plasma C-reactive protein (CRP) level with bronchopulmonary dysplasia (BPD).

Variable^*^	BPD cases/controls, *n*	BPD cases/controls with CRP above median, *n* (%)^†^	OR (95% CI)^‡^	*p* ^‡^
Maximum CRP	73/202	53 (72.6)/82 (40.6)	3.40 (1.76–6.58)	3.0 × 10^−4^
Mean CRP	73/202	54 (74.0)/84 (41.6)	3.57 (1.88–6.77)	9.7 × 10^−5^

*Definition of abbreviations*: BPD, bronchopulmonary dysplasia; CI, confidence interval; CRP, C-reactive protein; OR, odds ratio.

^*^Maximum or mean CRP level (mg/L) during the first week of life. In Finland, a CRP level of <3 mg/L is considered to be within the normal reference range, but no normative values for preterm infants are available.

^†^ *χ* ^2^ test *p* values were 3.0 × 10^−6^ and 2.0 × 10^−6^ for maximum and mean CRP, respectively.

^‡^Logistic regression with gestational age and small-for gestational (SGA, defined as birthweight Z-score of ≤−2 SD) as covariates. Odds ratio given for maximum and mean plasma CRP (above/below median) during first week of life.

In LR analysis with GA and SGA as covariates, both the maximum CRP level during the first week and the mean CRP level during the first week were significant predictors of BPD, with ORs of 3.4 and 3.6, respectively (Table 5).

### SNP rs11265269 in the *CRP* gene region is an independent risk factor of BPD

To consolidate the evidence on the role of rs11265269 as a risk factor of BPD, this SNP was analysed together with the significant antenatal risk factors, GA and SGA. Table 6 shows the results of LR analysis that included rs11265269 in the model. This SNP predicted the risk of BPD in each population, independently of the degree of prematurity and SGA status.

**Table 6:** Logistic regression model describing predictors of bronchopulmonary dysplasia.

Variable	GWAS		Internal replication populations joint		All Finnish populations joint	
	OR (95% CI)	*p*	OR (95% CI)	*p*	OR (95% CI)	*p*
SNP rs11265269	3.37 (1.89–6.00)^*^	3.76 × 10^–5^	1.45 (1.02–2.05)^*^	0.038	1.82 1.36–2.43)^*^	5.32 × 10^−5^
Gestational age^†^	0.83 (0.68–1.02)	0.076	0.67 (0.60–0.75)	5.31 × 10^−12^	0.70 (0.63–0.77)	2.67 × 10^−13^
SGA^‡^	3.50 (1.58–7.76)	1.98 × 10^–3^	2.65 (1.64−4.26)	6.45 × 10^−5^	2.86 (1.91–4.28)	3.05 × 10^−7^

*Definition of abbreviations*: GWAS, genome-wide association study; CI, confidence interval; OR, odds ratio; SNP, single-nucleotide polymorphism; SGA, small for gestational age.

^*^Odds ratio for minor allele under additive model.

^†^As continuous variable in the model.

^‡^Defined as birthweight Z-score of ≤−2 SD. Birthweight Z-score describes distribution of birthweight at given length of gestation in SD.

## Discussion

BPD is a multifactorial disease with high heritability. We conducted a GWAS on a relatively genetically homogenous Finnish population and observed a suggestive association between moderate-to-severe BPD and SNP rs11265269 near the *CRP* gene. This association was replicated in an independent Finnish population and in population of infants of African descent from France. Furthermore, plasma levels of CRP during the first week of life predicted the development of BPD. Additionally, the known antenatal risk factors and rs11265269 additively predisposed to BPD. Finally, another SNP from the *CRP*-gene region, SNP rs3093059, associated with plasma CRP levels. No significant association was noted with the SNP rs11265269 and plasma CRP, however.

CRP is a prominent clinical marker of infections and inflammatory disease. Persisting inflammation is a major finding in infants developing BPD. Mechanical ventilation and hyperoxia are known postnatal risk factors for BPD and have been shown to induce inflammation^24, 25^. CRP is mainly produced in the liver. One of the main ligands of CRP is phosphocholine, a constituent of many bacterial and fungal polysaccharides. Phosphocholine is also present in dead or dying cells^26^. IL-6 and IL-1 have a major synergistic effect on the induction of CRP expression. High level of CRP activates phagocytic cells, stimulates production of inflammatory cytokines, regulates the classical complement pathway and may cause adverse vascular events when present in excess^27^.

The plasma levels of CRP are generally low at birth^28^, and a mechanism of deficient CRP synthesis in fetal liver has been proposed^29^. In present study, plasma levels of CRP before age 12 hours were mostly low and predicted neither BPD nor sepsis. By contrast, it was the increase in plasma CRP after birth and the maintenance of variably elevated CRP levels during the first week of life that discriminated between infants who will develop BPD and those who will not. This is in line with what earlier has been reported about CRP and BPD, namely that CRP on day 28 and BPD associated^30^, and that there was a qualitative association between CRP levels from days 0 to 21 and BPD^31^. In present study, the association between CRP values and BPD was demonstrated already during the first week of life, which is of clinical importance. Our novel finding, that elevated plasma CRP values from the first week of life can be used as a biomarker for an increased risk of BPD, needs to be confirmed in independent studies. The clinical feasibility of CRP makes it superior to the other markers identified with an increased risk of BPD^32^. Noteworthy the CRP values did not correlate with either early- or late-onset sepsis. This is in consonance with what is known about CRP in the preterm i.e. that both the baseline level of CRP and the response to infection are lower in premature infants compared to the term new-borns. Also the sensitivity and specificity of CRP in diagnosing sepsis are lower at preterm neonates compared to their term peers^33^. As all other laboratory values, the interpretation of CRP requires the context of other findings and clinical signs of the patient.

Besides inflammation, infection and other environmental factors, genetic polymorphisms influence the expression of CRP. The heritability of plasma CRP is estimated to be 25–40%, and levels of CRP vary among different ethnic groups^34, 35^. Moreover, the SNPs reported to affect CRP levels vary among different populations^23, 36^. As such, the lack of an association between rs11265269 and BPD in one of the external replication populations is not surprising. CRP response varies also between species and for instance in mice *Crp* has generally low expression levels^37^, thus complicating further confirmation of the findings of this study.

A strength of this study is the relative homogeneity of the population in the GWAS and in the internal replication sets. Because of the Finnish population history with a restricted number of founders, the allelic diversity in Finland is lower than in most other populations^38^. This compensates for the main limitation of the study, namely the small sample size in the GWAS. However, it is possible that the small sample size limits the overall generalization of our GWAS findings to other study populations. Another limitation is the lack of association between rs11265269 and plasma CRP levels. This SNP, however, is located in the same LD region with rs3093059 and other *CRP*-associated SNPs, and also haplotypes within the region associated with BPD. We propose that rs11265269 may show milder association with CRP plasma levels that remains undetected in our set of study subjects, or it may associate with the CRP synthesis in lung cells^39^. Unfortunately, potentially valuable data on expression of *CRP* in lung compartments was not available. Further credence to association between rs11265269 and moderate-to-severe BPD is given by the finding that the association remained similar when infants with mild BPD were excluded from the controls. Furthermore, as altered treatment practices and changes in diagnostics may have influenced the results, we repeated the analyses in subgroups of infants born in two time periods (before and after 2010) and the association remained similar. It is also possible that the potential predisposing role of rs11265269 in BPD is linked to a gene other than *CRP*, such as *DUSP23*, the adjacent gene, encoding dual specificity phosphatase 23. DUSP23 is a phosphatase involved in regulation of cell-cell adhesion^40^. Its potential link to BPD remains to be investigated.

Although none of the initial GWAS signals were consistently replicated in our replication populations, some of these may represent real association signals. As an example, of the SNPs suggestively associated with BPD in GWAS, those within *RASGFR1* (encoding Ras protein specific guanine nucleotide releasing factor 1) may represent real associations, since other SNPs located near this gene showed association signals in a previous GWAS of BPD^11^. Furthermore, some of the other genes with suggestively associated SNPs could be biologically relevant for the BPD phenotype. As an example, variants near the *SGCD* gene (encoding sarcoglycan delta) are known to be associated with airway responsiveness, a process that is linked to decline in lung function in subjects with COPD^41^. Larger studies are required to assess the potential role of these genes in BPD.

Although the heritability of BPD is estimated to be high, consistent and statistically significant associations have not been detected in genomic studies. Current evidence suggests a multiplicity of genes and pathways involved in the pathogenesis of BPD. Besides GWAS, these pathways have been suggested in other large-scale studies, including exome sequencing and transcriptomic studies. It is possible that rare predisposing variants detected by exome sequencing can explain part of the missing heritability of BPD; these rare variants would be missed with the GWAS strategy. In line with our present finding, *CRP* was one of the top five genes characterizing severe BPD compared to non-BPD infants in a recent exome sequencing study^42^. In another exome sequencing study, Li *et al*. tentatively identified 258 genes with rare nonsynonymous variants in patients with moderate-to-severe BPD. These genes represented pulmonary structure and function, morphogenesis of embryonic epithelium, and regulation of the Wnt signalling pathway^43^. On the other hand, Ambalavanan and co-workers combined a GWAS with pathway analysis and found evidence suggesting involvement of both known (phosphorus oxygen lyase activity) and new (targets of miR-219) pathways in the pathophysiology of BPD^12^. Bhattacharya *et al*. performed a genome-wide gene-expression study and reported evidence on accumulation of connective tissue inflammatory mast cells in the lungs of infants who died of BPD^44^. In addition to further exome and whole-genome sequencing studies to detect rare predisposing variants, additional approaches, such as meta-analyses, pharmacogenomics and experimental studies are required. Identification of BPD-predisposing genes would bring information about the pathogenesis of BPD, ultimately enabling discovery of new individualized strategies against this complex syndrome.

We discovered a suggestive association between rs11265269 polymorphism near *CRP* gene and the risk of BPD. Plasma CRP shortly after birth predicts the risk of BPD. Together with the known antenatal risk factors rs11265269 influences susceptibility to BPD. As such, we propose that genetic and environmental factors influence the expression of *CRP* that may stimulate pathways predisposing to BPD.

## Electronic supplementary material

Supplementary Information
