# Supplementary material for: Genome-wide association study of bronchopulmonary dysplasia: a potential role for variants near the CRP gene
Source: Sci Rep. 2017 Aug 24;7:9271. doi: 10.1038/s41598-017-08977-w (PMC5571168; doi:10.1038/s41598-017-08977-w)
Supplement: Supplementary file 1 — Supplementary Information [file 41598_2017_8977_MOESM1_ESM.pdf]

Supplementary Information

Genome-wide association study of bronchopulmonary dysplasia: a potential role for variants near the *CRP* gene

Mari Mahlman[+*], Minna K. Karjalainen[+], Johanna M. Huusko, Sture Andersson, M. Anneli Kari, Outi K.T. Tammela, Ulla Sankilampi, Liisa Lehtonen, Riitta H. Marttila, Dirk Bassler, Christian F. Poets, Thierry Lacaze-Masmonteil, Claude Danan, Christophe Delacourt, Aarno Palotie, Louis J. Muglia, Pascal M. Lavoie, Alice Hadchouel, Mika Rämet[†], Mikko Hallman[†]

[+] These authors contributed equally to this work.

[†] These authors contributed equally to this work.

SUPPLEMENTARY TEXT FOR METHODS

Study Populations

The infants for the discovery GWAS analysis (60 cases and 114 controls) and for the two internal replication analyses (136 cases and 419 controls) were of Finnish origin. The discovery population was collected in the Finnish University Hospitals during 1997-2012, and infants in the internal replicates in 1997-2015. A subset of these infants have been included in our previous candidate gene studies[1-4], while the rest were from a subsequent prospective sample collection. We selected the infants for GWAS and the first internal replicate using the matching criteria described below. A subset of the populations analyzed in the GWAS and first internal replication populations were studied for plasma CRP levels; infants for whom CRP levels could be recorded were included (n = 275; 112 infants from GWAS, 163 infants from the first internal replicate).

The Canadian infants for the external replication analysis (50 cases and 51 controls) were of Caucasian origin, and data were collected from neonatal intensive care units as described previously[5]. The French infants, also for the external replication analysis, were of either Caucasian (48 cases and 163 controls) or African (25 cases and 51 controls) origin; their ethnicity was defined based on the parental origin. The Canadian and French infants of Caucasian origin were pooled for analyses (and are referred to as external replication population 1). Sixty-four percent of the French infants represented the population described previously by Hadchouel et al.[6]; the rest corresponded to samples collected retrospectively from former preterm infants born in 1997 (Table S2). The Canadian infants were selected out of the 269 infants in a previous study[5], on the basis of the Caucasian ethnicity, the present inclusion criteria and the availability of DNA (Table S2).

The oxygen reduction test[7] was used for infants born in Finland after 2009. Of the total study subjects, 42.1% were born in 2009 or later and 57.9% were born between 1997 and 2008.

Selection of study patients

To augment the power of the study, two control infants were chosen for each infant with BPD. To limit the effect of known antenatal risk factors for BPD, an effort was made to match control infants with case infants according to (1) gestational age (GA), (2) small-for-gestational-age (SGA) status and 3) male gender. Matching was done only for the Finnish study infants (GWAS and internal replication 1) and led to statistical similarity only for GA and male gender in the GWAS. No matching was done for the second internal replication population and external replication populations. Only infants surviving for more than 36 postconceptional weeks were included. Only one infant was selected from sibling pairs. Selection criteria for case infants from multiple pregnancies were (1) the most affected infant and (2) the nonpresenting infant, if there was no difference in BPD status.

DNA sample preparation and genotyping

DNA samples were obtained from umbilical cord blood or tissue, buccal cells, saliva, or tracheal aspirate. For details regarding DNA sample preparation, see Table S3. Genome-wide genotyping and targeted SNP genotyping were performed with the Infinium HumanCoreExome BeadChip (Illumina, San Diego, CA, USA) and iPLEX Gold (Sequenom, San Diego, CA, USA) assays, respectively, by the Technology Centre, Institute for Molecular Medicine Finland (FIMM), University of Helsinki. SNPs rs11265269, rs1889268 and rs2536512, included in the final step of the study, were genotyped using PCR restriction fragment length polymorphism analysis with the restriction enzymes HpyCH4III, FokI, and BssHII (New England Biolabs, Ipswich, MA, USA). Genotypes of SNP rs599915 were determined by sequencing.

Data processing, imputation, and selection of SNPs for replication analyses

Quality control for genome-wide SNP data. Processing of genome-wide data was performed with PLINK, v. 1.07[8]. SNPs with minor allele frequency (MAF) < 0.01 or genotyping failure > 0.1 and

those deviating from Hardy–Weinberg equilibrium ($p < 0.001$) were excluded. Two individuals were excluded as population outliers based on identical by descent (IBS) clustering and multidimensional scaling (MDS) analysis performed on a linkage disequilibrium–pruned autosomal SNP set (window size of 50 SNPs and variance inflation factor of 2 for SNPs with MAF < 0.05). MDS and quantile–quantile (QQ) plots were drawn with R (https://www.r-project.org). After quality control, 276,306 SNPs with MAF > 0.01 and 60 cases with moderate-to-severe BPD and 114 controls remained for analysis.

Imputation. For imputation of genotypes, i.e., statistical prediction of nongenotyped SNPs, genome-wide SNP data were prephased with SHAPEIT2[9], followed by imputation with IMPUTE2[10] using the 1000genomes phase 1 reference panel (Dec 2013 release).

Selection of SNPs for replication analyses. SNPs that fulfilled the following criteria in the GWAS were selected for further genotyping in the first internal Finnish replication population: (1) A p of $<5 \times 10^{-4}$ (i.e., $-\log_{10}(p) > 3.3$) in primary association analysis (99 SNPs, Table S4), (2) *CRP* gene region tagging SNPs or SNPs previously known to be associated with CRP levels (13 additional SNPs, Table S11; this criterion was applied because the top SNP in the GWAS was located near *CRP*), (3) SNPs previously shown to be associated with BPD showing allele frequency difference > 0.05 (12 SNPs, Table S8) or any SNP within 20 kb region of a previously associated SNP with $p < 0.05$ (31 additional SNPs, Table S9). To select these SNPs, we screened previously associated SNPs and genes from published genetic studies of BPD, including the GWASs by Hadchouel et al.[6] and Wang et al.[11]. Because the GWAS by Ambavanan et al.[12] was published after we had selected the SNPs for replication analyses, SNPs that were suggestively associated with BPD in this study were not analysed in our replication set. If any pair of SNPs selected according to the criteria was correlated ($r^2 \geq 0.8$), only one of each SNP pair was included. A subset of the original SNPs (seven SNPs) was replaced by tagging SNPs (determined from the 1000genomes Finnish population, http://www.1000genomes.org) if they otherwise did not settle in the genotyping sets. Some of the

SNPs fulfilling the selection criteria did not settle in the IPLEX genotyping sets and could thus not be analyzed in the replication populations; these SNPs are indicated in Tables S4 and S9.

In the external replication population, SNPs were chosen for genotyping based on the following criteria: (1) SNPs that showed allele frequency differences in the same direction as in the GWAS in the internal replication population and with $p < 0.01$ with the populations combined (31 SNPs, Table S5), (2) Previously associated SNPs or SNPs near previously associated genes with $p < 0.05$ with the populations combined and MAF difference in the same direction in the internal replication population (seven SNPs, Table S10), (3) SNPs in the *CRP* region with $p < 0.05$ with the populations combined (three SNPs, Table S12). SNPs with $p < 0.05$ in the Caucasian or French African replication populations (rs11265269, rs1889268, rs599915, and previously BPD-associated rs2536512) were genotyped in the final step of the study.

Statistical analyses

Significance level. Due to testing of multiple SNPs, the significance level in a GWAS is extremely strict. With 276,306 SNPs tested in the GWAS, the genome-wide significance level was set to $p < 1.8 \times 10^{-7}$ (i.e., $-\log_{10}(p) > 6.74$) under the conservative Bonferroni correction. Because we focused our analyses on the genotyped set, this represents an appropriate correction. Overly strict multiple-testing correction, however, can lead to exclusion of real associations; a SNP with $p < 10^{-5}$ in GWAS can be considered to be suggestively associated[13, 14]. To further avoid excluding potential false-negatives, we included SNPs with an even less stringent significance level ($p < 5 \times 10^{-4}$) in our replicate analyses. For the first internal (155 SNPs) and external (41 SNPs) replication sets, the multiple-comparison corrected significance levels were $p < 3.2 \times 10^{-4}$ and 1.2×10^{-3}, respectively, under the Bonferroni correction.

Association analysis. To compare allele frequencies of SNPs in cases and controls PLINK,[8] v. 1.07 or 1.09 was used. Because there was no difference in GA between BPD cases and controls in the

discovery population analysed in the GWAS, GWAS analyses were performed using basic association analysis (X^2 test). For analyses of the other populations, logistic regression with GA as a covariate was used. SNP–SNP interactions were analysed using the *epistasis* option in PLINK. Haplotype analyses were performed with Haploview, v. 4.2[15].

Association of plasma CRP levels during the first week of life with genotypes and risk of BPD. Available laboratory data about concentrations of plasma CRP during the first week of life were collected for 275 preterm Finnish infants (a subset of the infants from populations analysed in the GWAS and in the first internal replication). The current practice of screening for congenital infection is to measure CRP levels daily during the first week of life. We defined the following two CRP markers: the highest CRP level and the mean CRP level, both during the first week of life. We used logistic regression to evaluate whether SNP genotypes were associated with binomial CRP markers (above/below median) using the number of surfactant doses as covariates. The number of surfactant (Poractant alfa) doses was included as a covariate in this analysis, because of the tested potential confounding factors this was the only variable associated significantly with CRP levels ($p < 0.001$) in the subset of infants analyzed for CRP levels. These analyses were performed with SPSS Statistics 20.0, IBM Corporation and PLINK, v. 1.09[8]. Associations of the incidence of BPD or SNP genotypes with CRP markers was assessed by the X^2 test or the nonparametric Kruskal–Wallis test with SPSS. We further used logistic regression to evaluate whether CRP levels are significant predictors of BPD using GA and SGA as covariates with SPSS.

TABLE S1. Clinical characteristics of internal replication study populations

Characteristics	1st Internal Replication			2nd Internal Replication		
	BPD cases	Controls	*p*	BPD cases	Controls	*p*
Total *n*	105	221		31	198	
Moderate/severe BPD, *n* (%)	54/51 (51.4/48.6)			14/17 (45.2/54.8)		
No BPD/mild BPD, *n* (%)		100/121 (45.2/54.8)			129/69 (65.2/34.8)	
GA, weeks*,†	26.91 ± 1.89 (23.3–30.7)	28.01 ± 1.60 (23.7–30.7)	4.2×10^{-7}	27,55 ± 2.26 (23.4–30.6)	28.89 ± 1.95 (23.0–30.9)	4.1×10^{-4}
GA < 28 wk, *n* (%)	73 (69.5)	93 (42.1)	4.0×10^{-6}	17 (54.8)	53 (26.8)	0.002
Birth weight, grams*	899 ± 298 (430–2300)	1089 ± 289 (370–1755)	8.1×10^{-8}	995 ± 397 (420–2060)	1210 ± 347 (550–2190)	0.001
Birth weight Z-score*,‡	−1.22 ± 1.46 (−4.6–4.3)	−0.85 ± 1.32 (−5.5–2.19)	0.024	−1.12 ± 1.93 (−4.0–4.4)	−0.90 ± 1.42 (−4.75–2.50)	0.285
Male gender, *n* (%)	65 (61.9)	114 (51.6)	0.08	21 (67.7)	94 (47.5)	0.036
Singletons, *n* (%)	85 (81.0)	179 (81.0)	0.99	26 (83.9)	156 (78.8)	0.515

Definition of abbreviations: BPD, bronchopulmonary dysplasia; GA, gestational age; SD, standard deviation.
*Mean ± standard deviation (range).
†GA defined on the basis of fetal ultrasound before 15 weeks of pregnancy.
‡Birthweight Z-score describes distribution of birthweight at given length of gestation in SD.

TABLE S2. Clinical characteristics of external replication study populations

Characteristics	Canadian cohort			French Caucasian cohort			French African cohort		
	BPD cases	Controls	*p*	BPD cases	Controls	*p*	BPD cases	Controls	*p*
Total *n*	50	51		48	163		25	51	
Moderate/severe BPD, *n* (%)	33/17 (66.0/34.0)			NA			NA		
No BPD/mild BPD, *n* (%)		23/28 (45.1/54.9)			NA			NA	
GA, weeks*	25.96 ± 1.62 (23–30)	27.75 ± 1.21 (24–29)	6.8×10^{-8}	26.37 ± 1.58 (24–30)	27.11 ± 1.60 (24–30)	5.4×10^{-4}	26.22 ± 1.23 (24–29)	26.47 ± 1.30 (24–30)	0.350
GA < 28 wk, *n* (%)	41 (82.0)	21 (41.2)	2.5×10^{-5}	39 (81.2)	108 (66.3)	0.047	23 (92.0)	48 (94.1)	0.726
Birth weight, grams*	831 ± 208 (450–1420)	1193 ± 230 (630–1900)	6.5×10^{-11}	828 ± 184 (530–1440)	991 ± 254 (520–1750)	3.0×10^{-5}	746 ± 175 (490–1220)	867 ± 221 (500–1410)	0.017
Male gender, *n* (%)	27 (54.0)	24 (47.1)	0.485	29 (60.4)	78 (47.9)	0.126	13 (52.0)	17 (33.3)	0.118
Singletons, *n* (%)	37 (74)	40 (78.4)		35 (72.9)	124 (76.1)	0.656	24 (96.0)	47 (92.2)	0.530

Definition of abbreviations: BPD, bronchopulmonary dysplasia; NA, information not available; GA, gestational age; SD, standard deviation.
*Mean ± standard deviation (range).

TABLE S3. DNA sample preparation

Source	Extraction method*	Whole-genome amplification	Sample purification for whole-genome amplification
Umbilical cord blood†‡	UltraClean DNA Blood Isolation Kit (MO BIO Laboratories Inc., Carlsbad, CA, USA)† Nucleon Kit (Amersham, GE Healthcare Sciences, Cardiff, UK)‡		
Umbilical cord tissue†§	Gentra Puregene Tissue Kit (Qiagen, Hilden, Germany)† ChargeSwitch gDNA Mini Tissue Kit (Invitrogen, Carlsbad, CA, USA)§		
Dried blood†	MOBIO UltraClean BloodSpin DNA Isolation Kit (MO BIO Laboratories)	Illustra GenomiPhi V2 DNA Amplification Kit (GE Healthcare Sciences, Cardiff, UK)	Illustra Microspin G-50 columns (GE Healthcare Sciences)
Buccal cells†	Chelex 100 (Bio-Rad, Hercules, CA, USA)	Illustra GenomiPhi V2 DNA Amplification Kit (GE Healthcare Sciences)	Illustra Microspin G-50 columns (GE Healthcare Sciences)
Saliva†‡	prepIT•L2P (DNA Genotek, Ontario, Canada)		
Blood§	QIAamp DNA Blood Midi Kit (Qiagen)		
Tracheal aspirate§	ChargeSwitch gDNA Buccal Cell Kit (Invitrogen)		

*Quality of DNA was evaluated by standard absorbance measurements (A260/280 and A260/230). Samples with low genotyping rate were excluded. Whole-genome amplified samples were subjected to strict quality control procedures as previously described[16].
†Samples collected in Finland.
‡Samples collected in France.
§Samples collected in Canada.

TABLE S4. SNPs with suggestive BPD associations in GWAS: results of GWAS and first internal replicate. SNPs are organized according to *p* value in GWAS. SNPs with $p < 0.0005$ in GWAS were analysed in the first internal replication population. SNPs selected for genotyping in external populations are highlighted in blue. SNPs with $p < 0.01$ with the GWAS and replication populations combined and minor allele frequency difference in the same direction in both populations were included.

SNP information				GWAS				Internal replication population 1				Joint analysis	
SNP	Chr	Position[*]	Genes[†]	Minor allele frequency in cases	Minor allele frequency in controls	OR[‡]	*p*	Minor allele frequency in cases	Minor allele frequency in controls	OR[§]	*p*	OR[§]	*p*
rs11265269	1	159,728,127	*CRP, DUSP23*	0.392	0.167	3.22	3.4×10^{-6}	0.278	0.230	1.42	0.097	1.90	9.0×10^{-5}
rs1481294[‖]	11	38,604,075	*LOC103312105, LOC105376635*	0.325	0.575	0.36	9.6×10^{-6}	-	-	-	-	-	-
rs2351857	7	137,467,829	*DGKI*	0.617	0.373	2.71	1.4×10^{-5}	0.371	0.427	0.80	0.208	1.22	0.140
rs11691168	2	74,999,114	*LOC102724482*	0.475	0.250	2.71	2.1×10^{-5}	0.283	0.219	1.30	0.219	1.82	2.0×10^{-4}
rs2149564	9	98,607,989	*LINC00476*	0.642	0.404	2.65	2.4×10^{-5}	0.474	0.473	0.91	0.613	1.38	0.024
rs6562965	13	77,351,486	*LOC105370265*	0.442	0.224	2.75	2.4×10^{-5}	0.260	0.247	1.03	0.897	1.49	0.011
rs11745686	5	74,198,611	*LOC105379039, FAM169A*	0.400	0.193	2.79	3.2×10^{-5}	0.243	0.232	1.20	0.406	1.63	2.4×10^{-3}
rs1403617	3	165,255,278	*LINC01322, BCHE*	0.475	0.254	2.65	3.2×10^{-5}	0.278	0.304	0.91	0.615	1.39	0.026
rs12788032	11	38,632,770	*LOC103312105, LOC105376635*	0.275	0.504	0.37	3.9×10^{-5}	0.426	0.443	0.99	0.941	0.69	0.010
rs1822471	15	79,327,227	*RASGRF1*	0.092	0.281	0.26	4.6×10^{-5}	0.194	0.243	0.81	0.326	0.58	2.4×10^{-3}
rs9552800	13	23,599,673	*LOC105370111, SGCG*	0.242	0.083	3.51	4.7×10^{-5}	0.122	0.117	1.02	0.953	1.67	0.012
rs17537018	5	155,458,803	*SGCD, LOC105377674*	0.367	0.171	2.81	4.7×10^{-5}	0.213	0.200	1.06	0.780	1.55	8.9×10^{-3}
rs2527506[**]	7	2,968,361	*CARD11*	0.325	0.140	2.95	4.8×10^{-5}	*tSNP rs2527507* 0.177	*tSNP* 0.193	*tSNP* 0.95	*tSNP* 0.81	-	-
rs4704970[‖]	5	155,500,992	*SGCD, LOC105377674*	0.358	0.167	2.79	5.8×10^{-5}	-	-	-	-	-	-
rs1358603	7	52,757,480	*LOC105375280, LOC101928257*	0.617	0.390	2.51	5.8×10^{-5}	0.474	0.443	1.06	0.755	1.42	0.012
rs4506388	23	130,210,648	*ARHGAP36*	0.534	0.281	2.94	6.1×10^{-5}	0.346	0.368	0.91	0.677	1.45	0.032
rs9979500[**]	21	48,029,698	*S100B, PRMT2*	0.450	0.241	2.57	6.6×10^{-5}	*tSNP rs9647249* 0.406	*tSNP* 0.359	*tSNP* 1.18	*tSNP* 0.355	-	-
rs4640066[‖]	13	77,354,747	*LOC105370265*	0.475	0.263	2.53	7.0×10^{-5}	-	-	-	-	-	-
rs2352931	16	86,169,068	*LOC105376778, LOC101928582*	0.200	0.412	0.36	7.0×10^{-5}	0.320	0.281	1.21	0.322	0.82	0.172
rs12603672	17	51,115,243	*LOC105371831, LOC101927337*	0.117	0.018	7.40	7.2×10^{-5}	0.045	0.035	1.42	0.431	2.82	1.6×10^{-3}
rs2279073	19	44,739,303	*ZNF227*	0.325	0.548	0.40	7.3×10^{-5}	0.445	0.516	0.76	0.128	0.58	2.0×10^{-4}
rs1044189[**]	12	7,053,149	*RNU7-1*	0.383	0.189	2.67	7.6×10^{-5}	*tSNP rs3794310* 0.211	*tSNP* 0.260	*tSNP* 0.76	*tSNP* 0.209	-	-
rs7934284	11	38,577,786	*LOC103312105, LOC105376635*	0.608	0.386	2.47	7.7×10^{-5}	0.450	0.384	1.26	0.219	1.64	7.4×10^{-4}
rs200642524[††]	8	10,470,709	*RP1L1*	0.067	0	NA	8.0×10^{-5}	0	0	-	-		
rs11200206	10	123,635,883	*ATE1*	0.442	0.237	2.55	8.3×10^{-5}	0.265	0.305	0.88	0.513	1.29	0.085
rs2543361	14	76,593,690	*GPATCHL2L, IFT43*	0.575	0.355	2.46	8.3×10^{-5}	0.465	0.399	1.21	0.281	1.52	2.3×10^{-3}
rs314277	6	105,407,662	*LIN28B*	0.275	0.110	3.08	8.4×10^{-5}	0.155	0.171	0.94	0.787	1.41	0.064
rs4583363	18	65,162,006	*DSEL, LOC643542*	0.408	0.211	2.59	9.2×10^{-5}	0.262	0.285	0.91	0.630	1.34	0.049

rs11178156	12	70635,747	*LINC01481, CNOT2*	0.258	0.474	0.39	9.7×10^{-5}	0.412	0.400	1.01	0.945	0.70	0.015
rs7534535	1	81,820,712	*ADGRL2*	0.475	0.268	2.48	1.0×10^{-4}	0.353	0.328	1.08	0.665	1.45	9.5×10^{-3}
rs10281877	7	12,819,414	*ARL4A, LOC105375157*	0.475	0.268	2.48	1.0×10^{-4}	0.328	0.357	0.90	0.568	1.32	0.060
rs3803236	13	111,117,745	*COL4A2*	0.300	0.518	0.40	1.0×10^{-4}	0.437	0.478	0.88	0.491	0.65	2.8×10^{-3}
rs3803232[‖]	13	111,119,296	*COL4A2*	0.300	0.518	0.40	1.0×10^{-4}	-	-	-	-	-	-
rs7154765	14	44,674,826	*LOC101927351, FSCB*	0.475	0.268	2.48	1.0×10^{-4}	0.310	0.334	0.86	0.438	1.27	0.104
rs6855600	4	96,918,295	*PDHA2, LOC105377338*	0.333	0.154	2.76	1.1×10^{-4}	0.216	0.197	1.28	0.277	1.71	1.8×10^{-3}
rs2266879	23	153,035,798	*PLXNB3*	0.227	0.474	0.33	1.2×10^{-4}	0.379	0.357	1.20	0.412	0.73	0.076
rs9521106	13	89,179,012	*LINC00433, LINC00560*	0.200	0.404	0.37	1.3×10^{-4}	0.380	0.342	1.27	0.191	0.78	0.096
rs2677468	2	15,6541,170	*LOC105373701, LOC105373702*	0.558	0.347	2.38	1.4×10^{-4}	0.379	0.405	0.90	0.549	1.28	0.075
rs6799773	3	26,815,224	*LRRC3B, LOC105377003*	0.475	0.272	2.42	1.4×10^{-4}	0.363	0.329	0.96	0.842	1.42	0.019
rs10946984[‡‡]	6	11,158,816	*SMIM13, NEDD9*	0.525	0.316	2.40	1.4×10^{-4}	0.433	0.395	1.15	0.421	1.47	4.8×10^{-3}
rs3735709	8	102,555,474	*GRHL2*	0.100	0.013	8.33	1.5×10^{-4}	0.010	0.035	0.30	0.121	1.66	0.185
rs10799746	1	22,625,246	*MIR4418, ZBTB40*	0.317	0.145	2.74	1.6×10^{-4}	0.232	0.225	1.04	0.864	1.46	0.021
rs10191977	2	11,881,039	*LPIN1*	0.542	0.333	2.36	1.7×10^{-4}	0.457	0.414	1.17	0.390	1.53	3.4×10^{-3}
rs788163	2	172,931,559	*METAP1D*	0.350	0.171	2.61	1.7×10^{-4}	0.248	0.228	1.02	0.908	1.43	0.023
rs11159448[‡‡]	14	80,606,203	*LOC105370591, LOC105370593*	0.192	0.061	3.62	1.8×10^{-4}	0.129	0.108	1.19	0.519	1.78	5.8×10^{-3}
rs1947771	16	77,888,266	*VAT1L*	0.292	0.500	0.41	1.9×10^{-4}	0.432	0.404	1.14	0.451	0.80	0.108
rs6792668	3	26,835,843	*LRRC3B, LOC105377003*	0.558	0.351	2.34	2.0×10^{-4}	0.434	0.417	0.92	0.628	1.32	0.050
rs139851878[††]	16	815,740	*MSLN*	0.059	0	-	2.0×10^{-4}	-	-	-	-	-	-
rs955949	2	76,918,322	*LRRTM4, LOC105374814*	0.617	0.408	2.34	2.1×10^{-4}	0.425	0.493	0.79	0.187	1.16	0.280
rs925404	2	79,982,689	*CTNNA2*	0.233	0.434	0.40	2.2×10^{-4}	0.407	0.373	1.06	0.741	0.78	0.071
rs2305161	2	177,134,172	*MTX2*	0.092	0.259	0.29	2.2×10^{-4}	0.158	0.184	0.81	0.385	0.56	3.3×10^{-3}
rs10859660	12	94,490,633	*PLXNC1, LOC105369911*	0.625	0.417	2.33	2.2×10^{-4}	0.436	0.440	0.87	0.447	1.26	0.095
rs10871454	16	31,048,079	*STX4*	0.533	0.329	2.33	2.2×10^{-4}	0.387	0.404	0.95	0.791	1.28	0.078
rs2876217	20	11,171,441	*LOC105372528, LOC339593*	0.433	0.241	2.41	2.2×10^{-4}	0.325	0.276	1.14	0.491	1.51	5.1×10^{-3}
rs12070002	1	227,819,490	*ZNF678*	0.583	0.377	2.31	2.4×10^{-4}	0.391	0.459	0.86	0.433	1.24	0.143
rs11619503	13	49,343,160	*CYSLTR2, FNDC3A*	0.217	0.079	3.23	2.4×10^{-4}	0.132	0.097	1.309	0.240	1.91	2.1×10^{-3}
rs1828037	18	68,530,960	*GTSCR1, LOC105376872*	0.258	0.461	0.41	2.4×10^{-4}	0.412	0.389	1.00	0.982	0.71	0.022
rs1777458	1	108,585,993	*VAV3, SLC25A24*	0.400	0.215	2.44	2.5×10^{-4}	0.287	0.268	1.15	0.499	1.60	3.0×10^{-3}
rs7672047	4	187,580,706	*FAT1*	0.275	0.478	0.41	2.5×10^{-4}	0.424	0.413	1.00	0.988	0.75	0.050
rs1482349	5	113,170,445	*LOC105379127, KCNN2*	0.658	0.452	2.34	2.5×10^{-4}	0.447	0.467	0.90	0.555	1.28	0.087
rs10985233	9	98,596,745	*LINC00476*	0.550	0.347	2.31	2.5×10^{-4}	0.407	0.423	0.89	0.483	1.24	0.126
rs10836987	11	38,477,555	*LOC103312105, LOC105376635*	0.292	0.496	0.42	2.5×10^{-4}	0.396	0.474	0.75	0.124	0.58	3.1×10^{-4}
rs6531132	2	19,082,713	*LOC105373456*	0.025	0.154	0.14	2.6×10^{-4}	0.063	0.125	0.54	0.069	0.34	2.2×10^{-4}
rs171350	2	54,924,896	*SPTBN1, EML6*	0.333	0.162	2.58	2.6×10^{-4}	0.184	0.193	0.96	0.863	1.43	0.034
rs6814283	4	111,388,265	*ENPEP, LOC105377362*	0.292	0.132	2.72	2.7×10^{-4}	0.181	0.181	0.97	0.908	1.45	0.035
rs1513765	4	139,193,151	*SLC7A11*	0.133	0.311	0.34	2.7×10^{-4}	0.197	0.251	0.71	0.115	0.57	1.4×10^{-3}
rs28538450	23	36,882,697	*LOC105373154*	0.227	0.070	3.90	2.7×10^{-4}	0.092	0.117	0.74	0.397	1.44	0.144
rs12023560	1	239,109,443	*LOC105373222, LOC102724174*	0.425	0.237	2.38	2.8×10^{-4}	0.266	0.280	0.83	0.342	1.26	0.126
rs67790212	4	13,305,522	*RAB28*	0.425	0.237	2.38	2.8×10^{-4}	0.240	0.290	0.76	0.183	1.24	0.163
rs749670[‖]	16	31,088,625	*ZNF646*	0.525	0.325	2.33	2.8×10^{-4}	-	-	-	-	-	-
rs8050894	16	31,104,509	*VKORC1*	0.525	0.325	2.30	2.8×10^{-4}	0.392	0.416	0.91	0.609	1.24	0.126
rs1889268	9	101,767,961	*COL15A1*	0.192	0.382	0.38	2.9×10^{-4}	0.283	0.339	0.73	0.120	0.61	1.9×10^{-3}
rs17878459	10	96,534,922	*CYP2C19*	0.083	0.008	10.27	2.9×10^{-4}	0.025	0.029	1.04	0.939	2.08	0.051
rs11707787	3	24,794,908	*LOC101927874, MIR4792*	0.267	0.115	2.80	3.0×10^{-4}	0.109	0.118	0.92	0.763	1.49	0.045

rs7732673[‡‡]	5	25,190,282	*LOC105374694*	0.392	0.211	2.41	3.0×10^{-4}	-	-	-	-	-	-
rs10424276[‖]	19	43,147,394	*LIPE-AS1*	0.017	0.136	0.11	3.1×10^{-4}	-	-	-	-	-	-
rs10413014	19	43,149,435	*LIPE-AS1*	0.017	0.136	0.11	3.1×10^{-4}	0.063	0.053	1.13	0.742	0.49	0.023
rs7114643	11	132,859,912	*OPCML*	0.375	0.197	2.44	3.2×10^{-4}	0.257	0.271	0.92	0.685	1.34	0.060
rs12056820	8	16,871,448	*FGF20, MICU3*	0.175	0.360	0.38	3.3×10^{-4}	0.260	0.256	1.13	0.568	0.72	0.045
rs4499340[‖]	19	43,152,051	*LIPE-AS1*	0.017	0.137	0.11	3.3×10^{-4}	-	-	-	-	-	-
rs4502159	15	50,468,959	*ATP8B4*	0.592	0.390	2.20	3.4×10^{-4}	0.414	0.521	0.62	8×10^{-3}	1.04	0.783
rs7224261	17	31,411,815	*ASIC2*	0.283	0.483	0.42	3.4×10^{-4}	0.413	0.388	1.13	0.488	0.82	0.166
rs4926244	19	13,374,913	*CACNA1A*	0.100	0.263	0.31	3.6×10^{-4}	0.151	0.189	0.71	0.152	0.52	1.1×10^{-3}
rs11107397[‖]	12	94,491,385	*PLXNC1, LOC105369911*	0.608	0.408	2.26	3.7×10^{-4}	-	-	-	-	-	-
rs6539212	12	105,848,626	*LOC105369956, LOC105369955*	0.442	0.254	2.32	3.7×10^{-4}	0.232	0.267	0.90	0.618	1.30	0.075
rs6695925	1	9,526,956	*LOC100506022, SLC25A33*	0.383	0.206	2.39	3.8×10^{-4}	0.273	0.254	1.03	0.887	1.44	0.016
rs4734731[§§]	8	104,834,911	*RIMS2*	0.233	0.425	0.41	3.8×10^{-4}	-	-	-	-	-	-
rs2716878	17	5,591,389	*LOC339166, NLRP1*	0.233	0.425	0.41	3.8×10^{-4}	0.337	0.313	1.15	0.472	0.77	0.090
rs664051	1	70,869,116	*HHLA3, CTH*	0.275	0.123	2.71	3.9×10^{-4}	0.193	0.194	0.95	0.809	1.32	0.093
rs7145153	14	80,684,865	*DIO2*	0.183	0.061	3.43	3.9×10^{-4}	0.121	0.115	0.98	0.928	1.57	0.037
rs1955625[‖]	14	80,726,296	*DIO2-AS1*	0.183	0.061	3.43	3.9×10^{-4}	-	-	-	-	-	-
rs2188014	8	16,862,202	*FGF20, MICU3*	0.367	0.193	2.42	4.0×10^{-4}	0.283	0.220	1.47	0.059	1.81	1.9×10^{-4}
rs2369194	10	30,028,267	*SVIL, LOC105376475*	0.350	0.180	2.46	4.0×10^{-4}	0.255	0.230	1.27	0.244	1.56	5.0×10^{-3}
rs799258[‡‡]	14	82,857,376	*LOC105370599, LOC101928599*	0.350	0.180	2.46	4.0×10^{-4}	-	-	-	-	-	-
rs12525800	6	12,035,329	*HIVEP1*	0.533	0.338	2.24	4.1×10^{-4}	0.420	0.389	1.12	0.531	1.40	0.018
rs9371388	6	150,625,469	*LOC105378054*	0.068	0.217	0.26	4.2×10^{-4}	0.297	0.303	0.95	0.782	0.73	0.058
rs11542374[‡‡]	8	144,886,809	*SCRIB*	0.017	0.132	0.11	4.2×10^{-4}	-	-	-	-	-	-
rs11787779	9	117,741,340	*TNC, TNFSF8*	0.108	0.272	0.33	4.2×10^{-4}	0.196	0.216	0.92	0.710	0.63	0.010
rs10469285[‖]	19	43,066,120	*LIPE-AS1*	0.017	0.132	0.11	4.2×10^{-4}	-	-	-	-	-	-
rs1126458[‖]	19	43,087,484	*CEACAM8, LIPE-AS1*	0.017	0.132	0.11	4.2×10^{-4}	-	-	-	-	-	-
rs12375076	7	49,848,808	*VWC2*	0.408	0.228	2.34	4.3×10^{-4}	0.287	0.249	1.26	0.227	1.53	4.1×10^{-3}
rs1031076	1	190,362,935	*BRINP3*	0.208	0.395	0.40	4.4×10^{-4}	0.345	0.356	0.90	0.589	0.72	0.030
rs7656124[**]	4	111,187,683	*ELOVL6, LOC105377362*	0.550	0.354	2.23	4.4×10^{-4}	*tSNP*<br>*rs7670205*<br>*0.385*	*tSNP*<br>*0.395*	*tSNP*<br>*0.93*	*tSNP*<br>*0.682*	-	-
rs913407	9	98,770,928	*ERCC6L2*	0.508	0.316	2.24	4.4×10^{-4}	0.359	0.380	0.87	0.428	1.22	0.150
rs3765166	2	17,269,3780	*SLC25A12*	0.392	0.215	2.35	4.5×10^{-4}	0.288	0.273	1.06	0.763	1.43	0.022
rs2490741	10	94,594,565	*EXOC6*	0.150	0.325	0.37	4.5×10^{-4}	0.278	0.267	1.03	0.887	0.73	0.055
rs7308147[‡‡]	12	70,680,393	*CNOT2*	0.198	0.071	3.25	4.5×10^{-4}	-	-	-	-	-	-
rs12582020	12	94,499,677	*PLXNC1, LOC105369911*	0.325	0.522	0.44	4.5×10^{-4}	0.485	0.490	0.89	0.532	0.79	0.095
rs4781487	16	13,555,023	*SHISA9*	0.392	0.215	2.35	4.5×10^{-4}	0.318	0.295	1.17	0.405	1.45	0.012
rs976603	3	76,842,705	*ROBO2*	0.300	0.496	0.44	4.6×10^{-4}	0.416	0.389	1.15	0.420	0.83	0.194
rs13159354	5	157,528,485	*LOC100128898, LOC105377677*	0.300	0.496	0.44	4.6×10^{-4}	0.424	0.498	0.72	0.074	0.60	5.8×10^{-4}
rs13155377[‖]	5	157,528,319	*LOC100128898, LOC105377677*	0.300	0.496	0.44	4.6×10^{-4}	-	-	-	-	-	-
rs10953831	7	116,571,673	*CAPZA2, ST7*	0.433	0.250	2.29	4.6×10^{-4}	0.355	0.273	1.37	0.086	1.66	6.3×10^{-4}
rs2417744	12	10,716,615	*LOC101060038*	0.267	0.118	2.71	4.6×10^{-4}	0.242	0.172	1.39	0.118	1.74	1.1×10^{-3}
rs11117451[‖]	16	86,163,221	*LINC01082, LOC101928582*	0.217	0.404	0.41	4.7×10^{-4}	-	-	-	-	-	-
rs889555[**]	16	31,122,571	*BCKDK*	0.183	0.364	0.39	4.8×10^{-4}	*tSNP*<br>*rs11865499*<br>0.287	*tSNP*<br>0.290	*tSNP*<br>0.99	*tSNP*<br>0.953	-	-

rs4822423	22	23,867,924	*LOC105372955, LOC105372954*	0.517	0.325	2.23	4.8×10^{-4}	0.475	0.401	1.31	0.145	1.60	1.2×10^{-3}
rs6690148	1	22,648,411	*MIR4418, ZBTB40*	0.417	0.237	2.30	5.0×10^{-4}	0.342	0.255	1.55	0.017	1.76	1.4×10^{-3}
rs5999125	22	34,263,325	*LARGE*	0.158	0.333	0.38	5.0×10^{-4}	0.223	0.277	0.75	0.164	0.58	1.1×10^{-3}

Definition of abbreviations: BPD, bronchopulmonary dysplasia; GWAS, genome-wide association study; OR, odds ratio; SNP, single-nucleotide polymorphism; tSNP, tagging single-nucleotide polymorphism.

[*]Chromosomal positions refer to human genome build 37 (GRCh37/hg19).

[†]Respective locus shown for SNPs within genes; two nearest loci shown for intergenic SNPs.

[‡]Odds ratio for minor allele in basic association analysis.

[§]Odds ratio for minor allele under additive model in logistic regression analysis with gestational age as a covariate.

[ll]SNP correlated with another SNP selected for replication analyses and was thus not genotyped.

[**]SNP was replaced by tagging SNP in replication analyses.

[††]SNP not polymorphic or very rare (minor allele frequency < 0.01) in replication population.

[‡‡]SNP did not settle in the IPLEX genotyping set for either the internal or external replicate and was thus not genotyped.

[§§]SNP deviated from Hardy–Weinberg equilibrium in replication population and was thus excluded.

TABLE S5. Results of SNPs analysed in the external replication populations. Allele frequency differences with $p < 0.1$ (with odds ratios in the same direction as in GWAS and in the first internal replication population) are highlighted in green. The SNPs rs1889268 (*COL15A1*) and rs5999125 (*LARGE*) are highlighted in red.

SNP information		External replication population 1 (Caucasian)				External replication population 2 (French African)			
SNP	Genes[*]	Minor allele frequency in cases	Minor allele frequency in controls	OR[†]	*p*	Minor allele frequency in cases	Minor allele frequency in controls	OR[†]	*p*
rs6690148	*MIR4418, ZBTB40*	0.191	0.221	0.95	0.817	0.208	0.180	1.31	0.573
rs7534535	*ADGRL2*	0.245	0.178	1.49	0.084	0.160	0.190	0.80	0.659
rs1777458	*VAV3, SLC25A24*	0.365	0.466	0.61	0.017	0.313[§]	0.382[§]	0.65[§]	0.321
rs11265269	*CRP, DUSP23*	0.263	0.259	0.90	0.629	0.440	0.235	2.48	0.017
rs10191977	*LPIN1*	0.429	0.428	1.05	0.813	0.375	0.529	0.48	0.066
rs6531132	*LOC105373456*	0.112	0.135	0.78	0.373	0.375	0.304	1.33	0.420
rs11691168	*LOC102724482*	0.199	0.235	0.81	0.327	0.479	0.390	1.43	0.323
rs2305161	*MTX2*	0.225	0.245	0.88	0.526	0.400	0.324	1.41	0.333
rs6855600	*PDHA2, LOC105377338*	0.189	0.270	0.63	0.047	0.320	0.302	1.11	0.782
rs1513765	*SLC7A11*	0.206	0.186	1.08	0.734	0.348[§]	0.490[§]	0.48[§]	0.084
rs11745686	*LOC105379039, FAM169A*	0.278	0.279	1.01	0.964	0.280	0.290	0.95	0.906
rs17537018	*SGCD, LOC105377674*	0.180	0.204	0.94	0.798	0.063	0.137	0.42	0.199
rs13159354	*LOC100128898, LOC105377677*	0.314	0.341	0.81	0.287	0.360[§]	0.382[§]	0.90[§]	0.785
rs12375076	*VWC2*	0.230	0.250	0.40	0.904	0.020	0.010	2.10	0.606
rs10953831	*CAPZA2, ST7*	0.258	0.301	0.83	0.389	0.140	0.069	2.41	0.145
rs2188014	*FGF20, MICU3*	0.270	0.265	1.05	0.822	0.020	0.059	0.29	0.263
rs1889268	*COL15A1*	0.291	0.351	0.65	0.038[‖]	0.271[§]	0.382[§]	0.56[§]	0.155
rs2369194	*SVIL, LOC105376475*	0.199	0.207	0.93	0.773	0.180	0.206	0.80	0.639
rs10836987	*LOC103312105, LOC105376635*	0.443	0.455	0.99	0.963	0.020	0.010	2.10	0.606
rs7934284	*LOC103312105, LOC105376635*	0.479	0.458	0.96	0.836	0.160[§]	0.100[§]	1.58[§]	0.356
rs2417744	*LOC101060038*	0.209	0.241	0.85	0.468	0.060	0.104	0.55	0.413
rs11619503	*CYSLTR2, FNDC3A*	0.051	0.044	1.29	0.545	0.360	0.304	1.31	0.477
rs3803236	*COL4A2*	0.390[‡]	0.378[‡]	1.13[‡]	0.524	0.380[§]	0.353[§]	1.11[§]	0.753
rs2543361	*GPATCHL2L, IFT43*	0.403	0.423	0.91	0.624	0.260	0.300	0.84	0.637
rs1822471	*RASGRF1*	0.190	0.177	1.00	0.995	0.479	0.430	1.24	0.545
rs12603672	*LOC105371831, LOC101927337*	0.066	0.063	1.06	1.000	0.160	0.108	1.55	0.398
rs4926244	*CACNA1A*	0.209	0.212	0.91	0.678	0.280	0.314	0.81	0.606
rs2279073	*ZNF227*	0.390[‡]	0.378[‡]	0.77[‡]	0.149	0.100	0.147	0.71	0.514
rs2876217	*LOC105372528, LOC339593*	0.449	0.498	0.77	0.149	0.280	0.255	1.12	0.759
rs4822423	*LOC105372955, LOC105372954*	0.479	0.498	0.91	0.598	0240[§]	0.290[§]	0.81[§]	0.578
rs5999125	*LARGE*	0.361	0.434	0.73	0.091[‖]	0.320	0.284	1.19	0.635

Definition of abbreviations: OR, odds ratio; SNP, single-nucleotide polymorphism; GWAS, genome-wide association study.

[*]Respective locus shown for SNPs within genes; two nearest loci shown for intergenic SNPs.

[†]Odds ratio for minor allele under additive model in logistic regression analysis with gestational age as a covariate.
[‡]Minor allele in Finnish populations is major allele in Caucasian external replication population. OR given for minor allele in Caucasian population.
[§]Minor allele in Finnish populations is major allele in African external replication population. OR given for minor allele in African population.
[‖]$p < 0.05$ when combined with the first Finnish internal replicate.

TABLE S6. Results of single-nucleotide polymorphisms rs1889268 (*COL15A1*) and rs5999125 (*LARGE*) analysed for association with bronchopulmonary dysplasia in two Finnish replication populations

SNP information		Internal replication population 1			Internal replication population 2			Internal replication populations joint			All Finnish populations joint		
SNP	Gene	Case / Control minor allele frequency	OR*	*p*	Case / Control minor allele frequency	OR*	*p*	Case / Control minor allele frequency	OR*	*p*	Case / Control minor allele frequency	OR*	*p*
rs1889268†	*COL15A1*	0.283 / 0.339	0.73	0.120	0.268 / 0.301	0.84	0.573	0.280 / 0.320	0.78	0.143	0.251 / 0.334	0.67	4.11×10^{-3}
rs5999125‡	*LARGE*	0.223 / 0.277	0.75	0.164	0.307 / 0.249	1.32	0.411	0.242 / 0.264	0.86	0.390	0.216 / 0.279	0.72	8.30×10^{-3}

Definition of abbreviations: BPD, bronchopulmonary dysplasia; OR, odds ratio; SNP, single-nucleotide polymorphism.
*OR for minor allele under additive model in logistic regression analysis with gestational age as a covariate.
†GWAS: case/control 0.192/0.382, OR 0.38, $p = 2.9 \times 10^{-4}$.
‡GWAS: case/control 0.158/0.333, OR 0.38, $p = 5.0 \times 10^{-4}$.

TABLE S7. Results of final three SNPs analysed for association with BPD without mild BPD infants among the controls. Association analysis was performed with moderate-severe BPD infants as cases (similarly to the primary analyses) and infants without BPD as controls, i.e. mild BPD infants were excluded from the controls.

SNP information		GWAS				Internal replication populations joint				All Finnish populations joint			
SNP	Gene	Minor allele frequency in cases	Minor allele frequency in controls without BPD	OR*	*p*	Minor allele frequency in cases	Minor allele frequency in controls without BPD	OR†	*p*	Minor allele frequency in cases	Minor allele frequency in controls without BPD	OR†	*p*
rs11265269	*CRP*, *DUSP23*	0.392	0.164	3.28	5.9×10^{-5}	0.277	0.225	1.48	0.079	0.313	0.212	1.79	1.4×10^{-3}
rs1889268	*COL15A1*	0.192	0.414	0.34	1.5×10^{-4}	0.280	0.331	0.65	0.043	0.251	0.350	0.57	1.4×10^{-3}
rs5999125	*LARGE*	0.158	0.297	0.45	9.6×10^{-3}	0.242	0.263	0.90	0.611	0.216	0.271	0.75	0.115

Definition of abbreviations: GWAS, genome-wide association study; OR, odds ratio; SNP, single-nucleotide polymorphism.
*Odds ratio for minor allele in basic association analysis.
†Odds ratio for minor allele under additive model in logistic regression analysis with gestational age as a covariate.

TABLE S8. SNPs previously associated or suggestively associated with BPD showing minor allele frequency differences of >0.05 in GWAS: results of GWAS and first internal replicate. SNPs selected for genotyping in external populations are highlighted in blue (SNPs with $p < 0.05$ with GWAS and replication populations combined).

SNP information					GWAS				Internal replication population 1				Joint analysis	
SNP	Chr	Position*	Location within gene	Evidence of previous association	Minor allele frequency in cases	Minor allele frequency in controls	OR†	*p*	Minor allele frequency in cases	Minor allele frequency in controls	OR‡	*p*	OR	*p*
rs2536512§	4	24,801,315	Exonic (A58T) within *SOD3*	Giusti et al 2012; Wang et al 2013ll	0.200	0.320	0.53	0.017	0.215	0.281	0.75	0.173	0.63	7.2×10^{-3}
rs5746136	6	160,103,084	Intronic within *SOD2*	Giusti et al 2012	0.317	0.368	0.79	0.336	0.282	0.322	0.86	0.433	0.84	0.242
rs7460507	8	10,969,075	Intronic within *XKR6*	Hadchouel et al 2011ll	0.283	0.355	0.72	0.175	0.350	0.339	1.04	0.842	0.89	0.446
rs10980199	9	112,858,901	Intronic within *PALM2-AKAP2*	Hadchouel et al 2011ll	0.075	0.171	0.39	0.014	0.106	0.127	0.87	0.627	0.63	0.046
rs1670137	10	67,727,223	Intronic within *CTNNA3*	Wang et al 2013ll	0.233	0.338	0.60	0.044	0.255	0.298	0.79	0.238	0.72	0.037
rs6480123	10	67,747,744	Intronic within *CTNNA3*	Wang et al 2013ll	0.208	0.316	0.57	0.034	0.237	0.265	0.85	0.423	0.74	0.060
rs4747046	10	72,273,552	Intronic within *KIAA1274*	Hadchouel et al 2011ll	0.433	0.487	0.81	0.342	0.510	0.486	1.01	0.945	0.95	0.694
rs10894472	11	131,756,065	Intronic within *HNT*	Hadchouel et al 2011ll	0.383	0.456	0.74	0.192	0.424	0.431	0.92	0.646	0.87	0.324
rs7174119	15	23,078,384	Intronic within *NIPA1*	Wang et al 2013ll	0.342	0.412	0.74	0.199	0.367	0.373	1.06	0.764	0.92	0.563
rs1474256	15	79,463,847	80 kb upstream of *RASGRF1*	Wang et al 2013ll	0.367	0.447	0.72	0.147	0.460	0.382	1.37	0.091	1.06	0.680
rs2701405	15	87,539,918	Intronic within *AGBL1*	Hadchouel et al 2011ll	0.367	0.421	0.80	0.325	0.414	0.438	0.95	0.773	0.91	0.495
rs16992771	19	5,046,070	Intronic within *JMJD2B*	Hadchouel et al 2011ll	0.442	0.377	1.31	0.243	0.422	0.453	0.86	0.383	1.00	0.981

Definition of abbreviations: BPD, bronchopulmonary dysplasia; MAF, minor allele frequency; GWAS, genome-wide association study; OR, odds ratio; SNP, single-nucleotide polymorphism.

*Chromosomal positions refer to human genome build 37 (GRCh37/hg19).

†Odds ratio for minor allele in basic association analysis.

‡Odds ratio for minor allele under additive model in logistic regression analysis with gestational age as a covariate.

§*SOD3* SNP was not associated with BPD in the second internal replicate (OR=1.13, *p*=0.697). OR was 0.76 (*p*=0.060) with all the Finnish populations combined.

llGenome-wide association study.

TABLE S9. SNPs located near previously associated genes showing nominal association ($p < 0.05$) with BPD in GWAS: results of GWAS and internal replicate. SNPs selected for genotyping in the external populations are highlighted in blue (SNPs with $p < 0.05$ with GWAS and replication populations combined and MAF difference of at least 0.03 in the replication population were selected).

SNP information					GWAS				Internal replication population				Joint analysis	
SNP	Chr	Position[*]	Location within gene	Evidence of previous association in gene	Minor allele frequency in cases	Minor allele frequency in controls	OR[†]	*p*	Minor allele frequency in cases	Minor allele frequency in controls	OR[‡]	*p*	OR[‡]	*p*
rs61761863[§]	1	169,677,923	Exonic (S49T) within *SELL*	Derzbach et al 2006	0.050	0.004	12.0	4.0×10^{-3}	-	-	-	-	-	-
rs3771166[§]	2	102,986,222	Intronic within *IL18R1*	Floros et al 2012	0.500	0.377	1.65	0.027	-	-	-	-	-	-
rs7663239[ll]	4	38,785,850	Upstream of *TLR10*	Mailaparambil et al 2010	0.008	0.053	0.15	0.038	*tSNP rs111812333 0.044*	*tSNP 0.039*	*tSNP 1.10*	*tSNP 0.841*		
rs3775580	4	185,383,746	Intronic within *IRF2*	Hadchouel et al 2011[**]	0.500	0.360	1.78	0.011	0.332	0.418	0.69	0.034	0.99	0.918
rs10980070	9	112,584,723	Intronic within *PALM2-AKAP2*	Hadchouel et al 2011[**]	0.158	0.079	2.20	0.022	0.081	0.063	1.30	0.435	1.63	0.045
rs10980082	9	112,606,655	Intronic within *PALM2-AKAP2*	Hadchouel et al 2011[**]	0.033	0.110	0.28	0.014	0.065	0.101	0.58	0.111	0.48	0.012
rs1981035	9	112,663,125	Intronic within *PALM2-AKAP2*	Hadchouel et al 2011[**]	0.125	0.215	0.52	0.040	0.201	0.196	1.04	0.862	0.81	0.233
rs1930249	9	112,735,377	Intronic within *PALM2-AKAP2*	Hadchouel et al 2011[**]	0.133	0.228	0.52	0.034	0.172	0.211	0.75	0.210	0.65	0.022
rs4978866[††]	9	112,735,585	Intronic within *PALM2-AKAP2*	Hadchouel et al 2011[**]	0.117	0.206	0.51	0.037	-	-	-	-	-	-
rs12555129	9	112,760,293	Intronic within *PALM2-AKAP2*	Hadchouel et al 2011[**]	0.283	0.189	1.70	0.043	0.163	0.190	0.89	0.610	1.16	0.397
rs10759387	9	112,787,897	Intronic within *PALM2-AKAP2*	Hadchouel et al 2011[**]	0.175	0.316	0.46	4.8×10^{-3}	0.232	0.242	0.94	0.772	0.73	0.052
rs260205	9	112,802,769	Intronic within *PALM2-AKAP2*	Hadchouel et al 2011[**]	0.500	0.364	1.75	0.014	0.437	0.416	1.03	0.877	1.25	0.115
rs2017392	9	112,820,620	Intronic within *PALM2-AKAP2*	Hadchouel et al 2011[**]	0.200	0.355	0.45	2.7×10^{-3}	0.284	0.298	0.95	0.802	0.74	0.050
rs552182[§]	9	112,825,422	Intronic within *PALM2-AKAP2*	Hadchouel et al 2011[**]	0.292	0.189	1.77	0.028	-	-	-	-	-	-
rs539817	9	112,830,219	Intronic within *PALM2-AKAP2*	Hadchouel et al 2011[**]	0.417	0.268	1.96	4h.6×10^{-3}	0.320	0.304	1.03	0.872	1.28	0.089
rs1980874	9	112,850,080	Intronic within *PALM2-AKAP2*	Hadchouel et al 2011[**]	0.092	0.193	0.42	0.014	0.102	0.137	0.79	0.374	0.59	0.016
rs10980199[‡‡]	9	112,858,901	Intronic within *PALM2-AKAP2*	Hadchouel et al 2011[**]	0.075	0.171	0.39	0.014	0.106	0.127	0.87	0.627	0.63	0.046
rs5030737	10	54,531,242	Exonic (C52R) within *MBL2*	Capoluongo et al 2007	0.025	0.079	0.30	0.045	0.030	0.040	0.69	0.465	0.48	0.060

rs1670137[‡‡]	10	67,727,223	Intronic within *CTNNA3*	Wang et al 2013[**]	0.233	0.338	0.60	0.044	0.255	0.298	0.79	0.238	0.72	0.037
rs6480123[§§]	10	67,747,744	Intronic within *CTNNA3*	Wang et al 2013[**]	0.208	0.316	0.57	0.033	-	-	-	-	-	-
rs200547164	10	73,767,254	Intronic (H155Q) within *CHST3*		0.042	0.004	9.87	0.011	-	-	-	-	-	-
rs737080 [llll]	10	73,820,911	Within *SPOCK2* 3´UTR	Hadchouel et al 2011[**]	0.008	0.057	0.14	0.028	0.035	0.044	0.71	0.480	0.48	0.077
rs932762[ll]	10	95,928,274	Intronic within *PLCE1*	Hadchouel et al 2011[**]	0.033	0.004	7.83	0.031	*tSNP rs11187798: 0.024*	*tSNP 0.014*	*tSNP 2.21*	*tSNP 0.242*	-	-
rs4265793 [llll]	16	19,028,549	Intronic within *TMC7*	Hadchouel et al 2011[**]	0	0.035	-	0.038	-	-	-	-	-	-
rs6564588	16	78,831,587	Intronic within *WWOX*	Hadchouel et al 2011[**]	0	0.048	-	0.014	0.040	0.041	0.81	0.628	0.49	0.081
rs1478457	19	23,065,269	Downstream of *ZNF723*	Wang et al 2013[**]	0.258	0.132	2.30	3.1×10^{-3}	0.188	0.140	1.34	0.223	1.67	4.6×10^{-3}
rs7882019	23	32,116,068	Intronic within *DMD*	Hadchouel et al 2011[**]	0.386	0.257	1.82	0.032	0.262	0.306	0.87	0.526	1.15	0.410
rs331369[§]	23	32,234,233	Intronic within *DMD*	Hadchouel et al 2011[**]	0.432	0.298	1.79	0.032	-	-	-	-	-	-
rs633	23	153,274,228	Downstream of *IRAK1*	Wang et al 2013[**]	0.068	0.164	0.37	0.031	0.186	0.149	1.26	0.417	0.93	0.756
rs145385375 [llll]	23	153,282,018	Exonic (E369G) within *IRAK1*	Wang et al 2013[**]	0.035	0	-	0.014	0	0	-	-		
rs1059702	23	153,284,192	Exonic (S196F) within *IRAK1*	Wang et al 2013[**]	0.034	0.140	0.22	8.0×10^{-3}	0.161	0.134	1.32	0.363	0.84	0.507

Definition of abbreviations: BPD, bronchopulmonary dysplasia; GWAS, genome-wide association study; MAF, minor allele frequency; OR, odds ratio; SNP, single-nucleotide polymorphism.
[*]Chromosomal positions refer to human genome build 37 (GRCh37/hg19).
[†]Odds ratio for minor allele in basic association analysis.
[‡]Odds ratio for minor allele under additive model in logistic regression analysis with gestational age as a covariate.
[§]SNP did not settle in IPLEX genotyping set and was thus not genotyped in replication analyses.
[ll]SNP was replaced by tagging SNP in replication analyses.
[**]Genome-wide association study.
[††]SNP correlated with another SNP selected for replication analyses and was thus not genotyped.
[‡‡]SNP also in Table S8.
[§§]SNP deviated from Hardy–Weinberg equilibrium in replication population and was thus excluded.
[llll]SNP not polymorphic or very rare (MAF < 0.01) in replication population.

TABLE S10. Summary of results for SNPs within or near genes previously associated with BPD analysed in external replication populations. Allele frequency differences with $p < 0.1$ are highlighted in green.

SNP information			GWAS				Internal replication 1 (Finnish)				External replication 1 (Caucasian)				External replication 2 (French African)			
SNP	Gene	Evidence of previous association in gene	Minor allele frequency in cases	Minor allele frequency in controls	OR*	*p*	Minor allele frequency in cases	Minor allele frequency in controls	OR†	*p*	Minor allele frequency in cases	Minor allele frequency in controls	OR†	*p*	Minor allele frequency in cases	Minor allele frequency in controls	OR†	*p*
rs2536512‡	*SOD3*	Giusti et al 2012	0.200	0.320	0.53	0.017	0.215	0281	0.75	0.173	0.309	0.360	0.70	0.072	0.167§	0.150§	1.10§	0.473
rs10980082	*PALM2-AKAP2*	Hadchouel et al 2011ll	0.033	0.110	0.28	0.014	0.065	0.101	0.58	0.111	0.117	0.129	0.98	0.952	0.042	0.078	0.49	0.388
rs1930249	*PALM2-AKAP2*	Hadchouel et al 2011ll	0.133	0.228	0.52	0.034	0.172	0.211	0.75	0.210	0.165	0.129	1.40	0.180	0.104	0.098	1.07	0.897
rs1980874	*PALM2-AKAP2*	Hadchouel et al 2011ll	0.091	0.193	0.42	0.014	0.102	0.137	0.79	0.374	0.240	0.247	0.94	0.747	0.500§	0.602§	1.54§	0.213
rs10980199	*PALM2-AKAP2*	Hadchouel et al 2011ll	0.075	0.171	0.39	0.013	0.106	0.127	0.87	0.628	0.160	0.185	0.81	0.396	0.432	0.418	1.04	0.897
rs1670137	*CTNNA3*	Wang et al 2013ll	0.233	0.338	0.60	0.044	0.255	0.298	0.79	0.628	0.155	0.136	1.08	0.761	0.200	0.167	1.31	0.543
rs1478457	*ZNF723*	Wang et al 2013ll	0.258	0.132	2.30	3.1×10^{-3}	0.188	0.140	1.34	0.137	0.163	0.170	0.95	0.848	0.520	0.450	1.26	0.499

Definition of abbreviations: BPD, bronchopulmonary dysplasia; GWAS, genome-wide association study; OR, odds ratio; SNP, single-nucleotide polymorphism.

*Odds ratio for minor allele in basic association analysis.

†Odds ratio for minor allele under additive model in logistic regression analysis with gestational age as a covariate.

‡ *SOD3* SNP was not associated with BPD in the second internal replicate (OR=1.13, *p*=0.697). OR was 0.76 (*p*=0.060) with all the Finnish populations combined.

§Minor allele in Finnish and Caucasian populations is major allele in African population. OR given for minor allele in African population.

llGenome-wide association study.

TABLE S11. SNPs in region surrounding *CRP*: results of GWAS and first internal replicate. SNPs from this region were selected for replication analyses so that most of the common variations in this region were tagged. Haplotype blocks from this region are shown in Figure 2, and association results for haplotypes are shown in Table 4. SNPs with $p < 0.05$ were selected for genotyping in external populations; these SNPs are highlighted in blue.

SNP information				GWAS				Internal replication population 1				Joint analysis	
SNP	Chr	Position*	Location relative to *CRP* gene	Minor allele frequency in cases	Minor allele frequency in controls	OR†	*p*	Minor allele frequency in cases	Minor allele frequency in controls	OR‡	*p*	OR‡	*p*
rs2794520§	1	159,678,816	Downstream	0.342	0.408	0.75	0.228	0.320	0.359	0.89	0.532	0.811	0.164
rs1800947§ ll	1	159,683,438	Exonic (synonymous, L184L) / intronic within *CRP* (in splice site)**	-	-	-	-	0.034	0.050	0.62	0.315	-	-
rs3093059§	1	159,685,136	Upstream	0.125	0.035	3.92	1.3×10^{-3}	0.069	0.057	1.45	0.315	2.12	6.5×10^{-3}
rs3122012	1	159,689,323	Upstream	0.317	0.342	0.89	0.632	0.337	0.368	0.84	0.332	0.86	0.304
rs2808635	1	159,694,209	Upstream	0.208	0.206	1.01	0.962	0.225	0.196	1.25	0.323	1.17	0.378
rs11265263	1	159,710,517	Upstream	0.142	0.044	3.60	1.2×10^{-3}	0.058	0.075	0.89	0.737	1.42	0.162
rs4285692	1	159,714,149	Upstream	0.442	0.417	1.11	0.654	0.421	0.379	1.20	0.298	1.17	0.245
rs4656849	1	159,723,521	Upstream	0.258	0.338	0.68	0.128	0.289	0.287	1.01	0.962	0.88	0.405
rs12094103	1	159,723,619	Upstream	0.342	0.487	0.55	9.5×10^{-3}	0.410	0.463	0.79	0.171	0.70	0.012
rs11265269	1	159,728,127	Upstream	0.392	0.167	3.22	3.4×10^{-6}	0.278	0.230	1.42	0.097	1.90	9.0×10^{-5}
rs4604689	1	159,736,139	Upstream	0.333	0.491	0.52	4.8×10^{-3}	0.393	0.396	1.00	0.988	0.80	0.121
rs7519478	1	159,743,845	Upstream	0.425	0.320	1.57	0.052	0.356	0.374	0.99	0.971	1.16	0.299
rs1129923	1	159,752,066	Upstream (within *DUSP23*)	0.183	0.105	1.91	0.041	0.102	0.134	0.63	0.097	0.96	0.858
rs4233356	1	159,753,183	Upstream	0.292	0.233	1.36	0.227	0.255	0.241	1.11	0.617	1.19	0.274

Definition of abbreviations: BPD, bronchopulmonary dysplasia; GWAS, genome-wide association study; OR, odds ratio; SNP, single-nucleotide polymorphism.

*Chromosomal positions refer to human genome build 37 (GRCh37/hg19).

†Odds ratio for minor allele in basic association analysis.

‡Odds ratio for minor allele under additive model in logistic regression analysis with gestational age as a covariate.

§SNP is known to be associated with serum CRP levels.

llSNP rs1800947 was not in GWAS chip; selected for replication analyses because this SNP is associated with CRP levels and is not tagged by any SNP.

** Depending on transcript variant.

TABLE S12. Summary of results for SNPs in region surrounding *CRP* analysed in external replication populations. Allele frequency differences with $p < 0.1$ are highlighted in green.

SNP information		GWAS				Internal replication 1 (Finnish)				External replication 1 (Caucasian)				External replication 2 (French African)			
SNP	Location relative to *CRP* gene	Minor allele frequency in cases	Minor allele frequency in controls	OR*	*p*	Minor allele frequency in cases	Minor allele frequency in controls	OR†	*p*	Minor allele frequency in cases	Minor allele frequency in controls	OR†	*p*	Minor allele frequency in cases	Minor allele frequency in controls	OR†	*p*
rs3093059	Upstream (~800 bp)	0.125	0.035	3.93	1.3×10^{-3}	0.069	0.057	1.45	0.320	0.102	0.086	1.18	0.600	0.354	0.265	1.46	0.300
rs12094103	Upstream (~40 kb)	0.342	0.487	0.55	9.5×10^{-3}	0.410	0.463	0.79	0.171	0.366	0.357	1.14	0.494	0.583‡	0.440‡	1.80‡	0.108
rs11265269§	Upstream (~44 kb)	0.392	0.167	3.22	3.4×10^{-6}	0.278	0.230	1.42	0.097	0.263	0.259	0.90	0.629	0.440	0.235	2.48	0.017

Definition of abbreviations: BPD, bronchopulmonary dysplasia; GWAS, genome-wide association study; OR, odds ratio; SNP, single-nucleotide polymorphism.
*Odds ratio for minor allele in basic association analysis.
†Odds ratio for minor allele under additive model in logistic regression analysis with gestational age as a covariate.
‡Minor allele in Finnish and Caucasian populations is major allele in African population. OR given for minor allele in African population.
§Results also shown also in Tables 3 and 4.

TABLE S13. Analysis of SNPs rs11265269 and rs3093059 with CRP levels in the first week of life. Suggestive associations ($p < 0.05$) are highlighted in green.

SNP	CRP level in each genotype group				$p^{\dagger}$	Logistic regression of CRP level[§] OR (95% confidence interval)[ll]	p
rs3093059	Genotype[*]	GG	GA	AA			
	n	1	34	234			
	Maximum CRP[‡]	15.0	12.6	10.4	0.308	2.00 (0.93-4.27)	0.074
	Mean CRP[‡]	7.0	6.2	4.7	0.210	2.19 (1.01-4.72)	0.046
rs11265269	Genotype[*]	GG	GA	AA			
	n	17	106	148			
	Maximum CRP[‡]	12.6	10.6	10.5	0.265	1.10 (0.72-1.69)	0.66
	Mean CRP[‡]	6.4	5.0	4.6	0.169	1.19 (0.77-1.83)	0.43

Definition of abbreviations: CRP, C-reactive protein; OR, odds ratio.
[*]G is the BPD-associated allele for both SNPs.
[†]Kruskal–Wallis test.
[‡]Mean of maximum or mean CRP level (mg/L) in the first week of life. In Finland, a CRP level of < 3 mg/L is considered to be within the normal reference range, but no normative values for preterm infants are available.
[§]Logistic regression performed under additive model for CRP divided into two classes (below/above median) with the number of surfactant doses (three classes: one, two, or more) as a covariate.
[ll]Odds ratio for minor allele G.

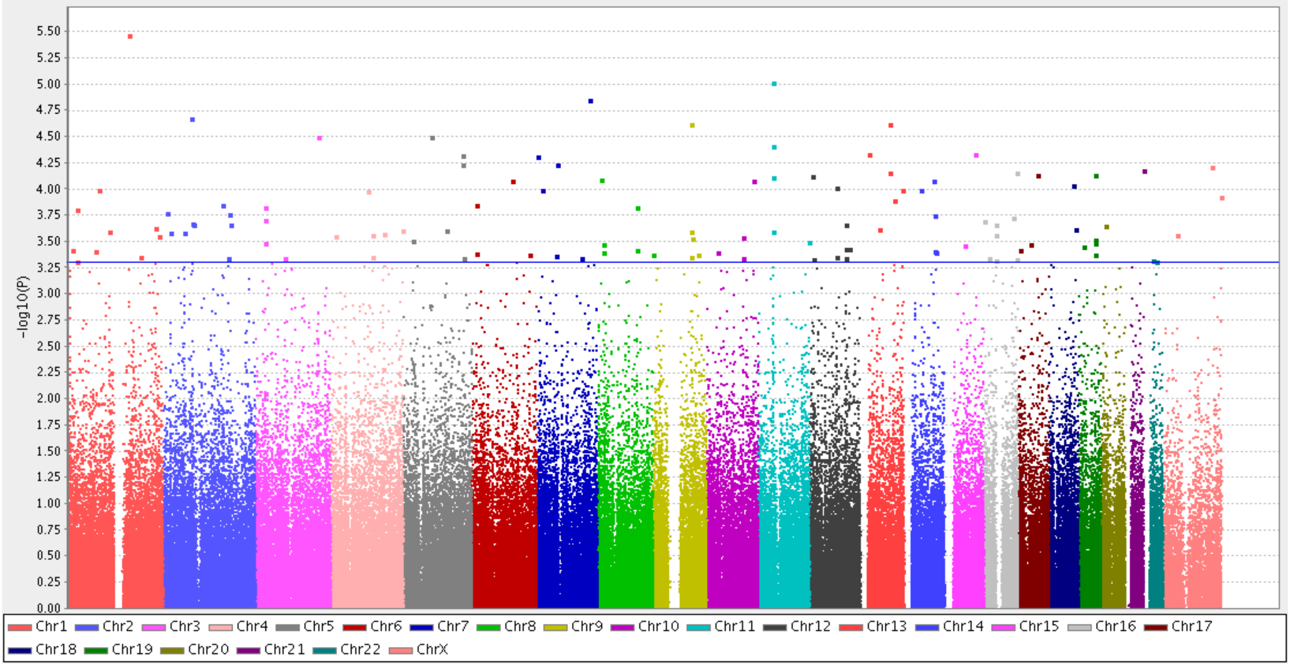

FIGURE S1. Manhattan plot summarizing results of GWAS of bronchopulmonary dysplasia (BPD). GWAS was performed for 276,306 SNPs with minor allele frequency > 0.01 in 60 cases with moderate-to-severe BPD and 114 controls. Each dot represents the $-\log_{10}(p)$ value of a single SNP analysed. None of the SNPs reached the genome-wide significance level ($-\log_{10}(p) > 6.74$). SNPs above the blue line ($-\log_{10}(p) > 3.3$; i.e., $p < 5 \times 10^{-4}$) were selected for further genotyping in the internal replication population. These SNPs are listed in Table S4.

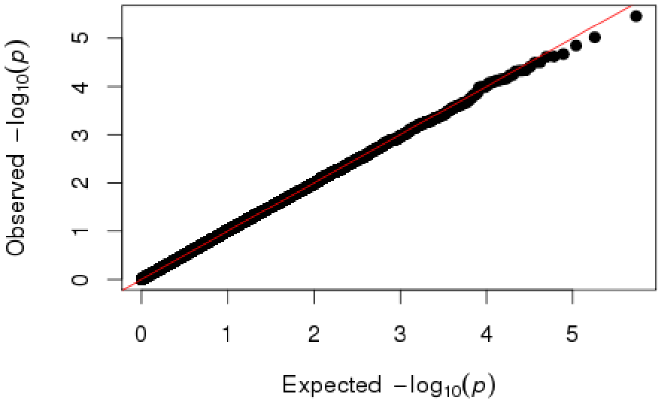

FIGURE S2. Quantile–quantile (QQ) plot for GWAS. Plot shows the expected $-\log_{10}(p)$ values under the hypothesis of no association versus the observed $-\log_{10}(p)$ values. As evinced by the QQ plot, population stratification was minimal ($\lambda = 1.004$).

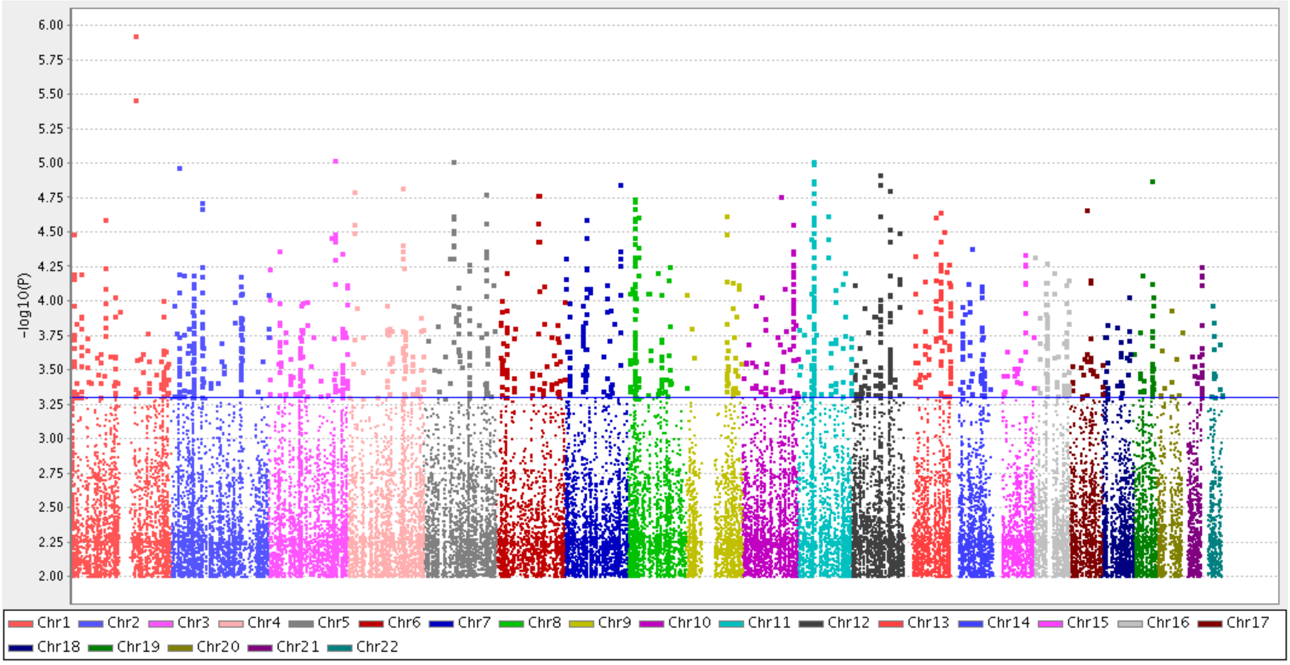

FIGURE S3. Manhattan plot of GWAS of bronchopulmonary dysplasia (BPD) including imputed SNPs. With imputed SNPs included for the autosomes, altogether 8,411,244 SNPs with minor allele frequency > 0.01 were studied in 60 cases with moderate-to-severe BPD and 114 controls. Each dot represents $-\log_{10}(p)$ value of a single SNP analysed. None of the SNPs reached the genome-wide significance level. SNPs for further genotyping in the internal replication population were selected from among the genotyped SNPs above the blue line ($-\log_{10}(p) > 3.3$; i.e., $p < 5 \times 10^{-4}$).

A)

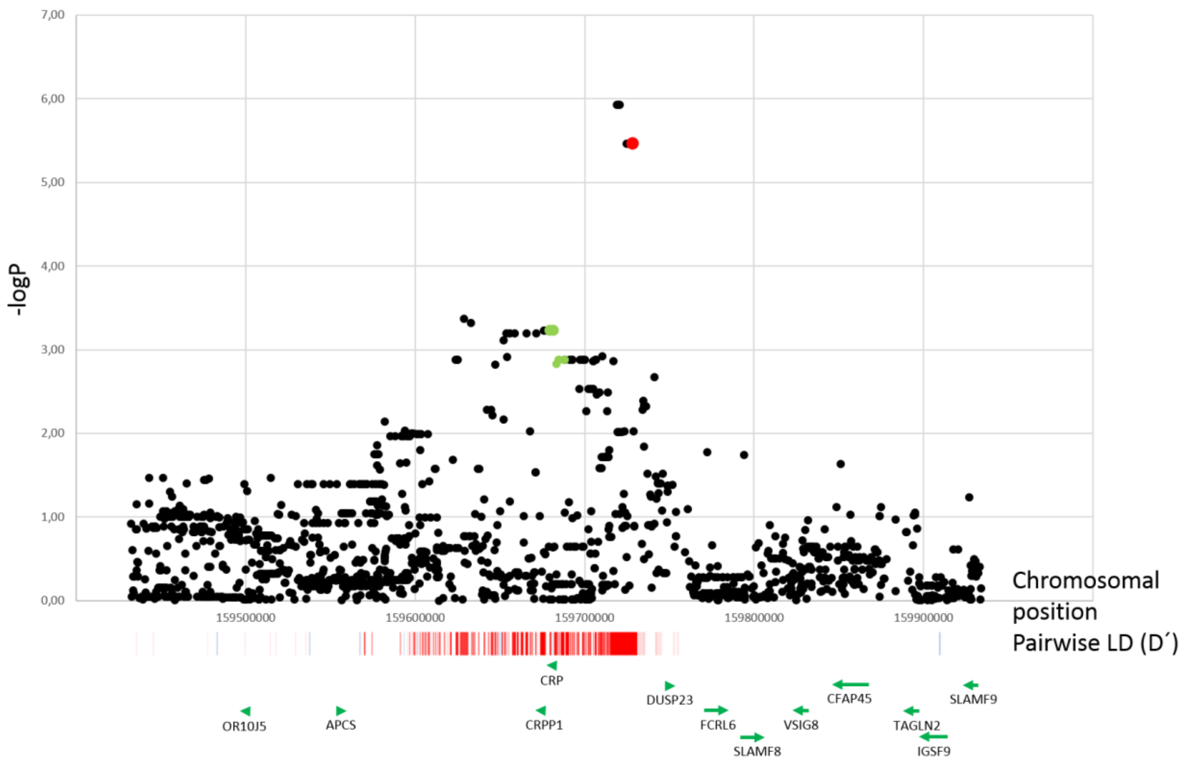

B)

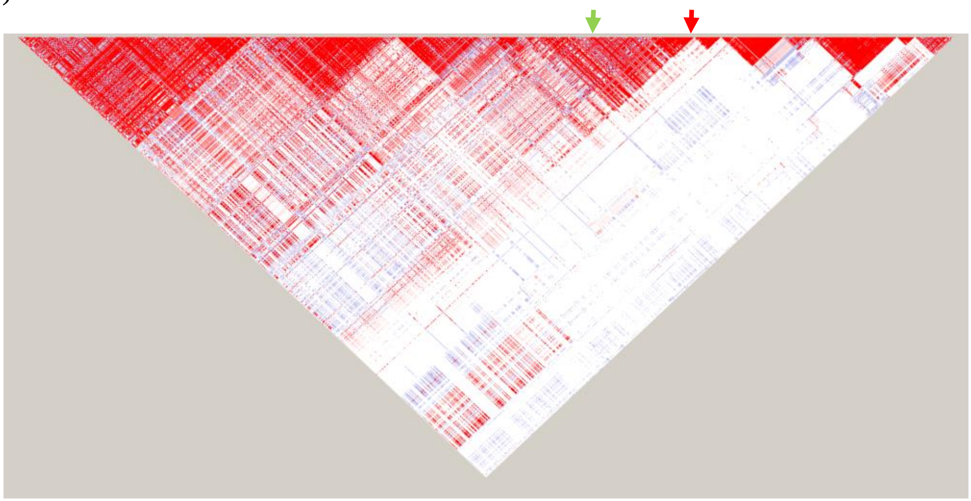

FIGURE S4. Detailed GWAS results for *CRP* gene region. (A) GWAS results for imputed SNPs on 500-kb region flanking *CRP*. Each dot represents $-\log_{10}(p)$ value of a single SNP analysed. SNP rs11265269 shown by red; SNPs within 5 kb of *CRP* gene showing association signals ($p < 0.05$) shown by green. Pairwise *D′* values of rs11265269 with SNPs in the region are visualized by different shades of red and pink; darker colors indicate stronger linkage disequilibrium (LD). Positions on chromosome 1 for human genome build 37 (GRCh37/hg19); genes shown below. B) LD plot for analysed SNPs in the region, including 948 SNPs with minor allele frequency > 0.05. Pairwise *D′* values for each SNP pair shown; darker colors indicate stronger LD. SNP rs11265269 (red arrow) is within a large LD region that extends to SNPs within and flanking *CRP*; these include several SNPs known to be associated with serum CRP levels, rs3093059 (green arrow) analysed in replication populations.

References

1. Rova, M. *et al*. Data mining and multiparameter analysis of lung surfactant protein genes in bronchopulmonary dysplasia. *Hum. Mol. Genet.* **13**, 1095-1104 (2004).

2. Huusko, J. M. *et al*. A study of genes encoding cytokines (IL6, IL10, TNF), cytokine receptors (IL6R, IL6ST), and glucocorticoid receptor (NR3C1) and susceptibility to bronchopulmonary dysplasia. *BMC Med. Genet.* **15**, 120-014-0120-7 (2014).

3. Huusko, J. M. *et al*. Polymorphisms of the gene encoding Kit ligand are associated with bronchopulmonary dysplasia. *Pediatr. Pulmonol.* (2014).

4. Mahlman, M. *et al*. Genes Encoding Vascular Endothelial Growth Factor A (VEGF-A) and VEGF Receptor 2 (VEGFR-2) and Risk for Bronchopulmonary Dysplasia. *Neonatology* **108**, 53-59 (2015).

5. Lavoie, P. M. *et al*. Influence of common non-synonymous Toll-like receptor 4 polymorphisms on bronchopulmonary dysplasia and prematurity in human infants. *PLoS One* **7**, e31351 (2012).

6. Hadchouel, A. *et al*. Identification of SPOCK2 as a susceptibility gene for bronchopulmonary dysplasia. *Am. J. Respir. Crit. Care Med.* **184**, 1164-1170 (2011).

7. Walsh, M. C. *et al*. Impact of a physiologic definition on bronchopulmonary dysplasia rates. *Pediatrics* **114**, 1305-1311 (2004).

8. Purcell, S. *et al*. PLINK: a tool set for whole-genome association and population-based linkage analyses. *Am. J. Hum. Genet.* **81**, 559-575 (2007).

9. Delaneau, O., Zagury, J. F. & Marchini, J. Improved whole-chromosome phasing for disease and population genetic studies. *Nat. Methods* **10**, 5-6 (2013).

10. Howie, B., Marchini, J. & Stephens, M. Genotype imputation with thousands of genomes. *G3 (Bethesda)* **1**, 457-470 (2011).

11. Wang, H. *et al*. A genome-wide association study (GWAS) for bronchopulmonary dysplasia. *Pediatrics* **132**, 290-297 (2013).

12. Ambalavanan, N. *et al*. Integrated Genomic Analyses in Bronchopulmonary Dysplasia. *J. Pediatr.* (2014).

13. Duggal, P., Gillanders, E. M., Holmes, T. N. & Bailey-Wilson, J. E. Establishing an adjusted p-value threshold to control the family-wide type 1 error in genome wide association studies. *BMC Genomics* **9**, 516-2164-9-516 (2008).

14. Zhang, Y. B. *et al*. Genome-wide association study identifies multiple susceptibility loci for craniofacial microsomia. *Nat. Commun.* **7**, 10605 (2016).

15. Barrett, J. C., Fry, B., Maller, J. & Daly, M. J. Haploview: analysis and visualization of LD and haplotype maps. *Bioinformatics* **21**, 263-265 (2005).

16. Karjalainen, M. K. *et al*. A potential novel spontaneous preterm birth gene, AR, identified by linkage and association analysis of X chromosomal markers. *PLoS One* **7**, e51378 (2012).
